# Evaluating the Potential and Accuracy of ChatGPT-3.5 and 4.0 in Medical Licensing and In-Training Examinations: Systematic Review and Meta-Analysis

**DOI:** 10.2196/68070

**Published:** 2025-09-19

**Authors:** Anila Jaleel, Umair Aziz, Ghulam Farid, Muhammad Zahid Bashir, Tehmasp Rehman Mirza, Syed Mohammad Khizar Abbas, Shiraz Aslam, Rana Muhammad Hassaan Sikander

**Affiliations:** 1 Shalamar Medical and Dental College Lahore Pakistan

**Keywords:** ChatGPT, medical education, accuracy of ChatGPT, ChatGPT-3.5 performance, ChatGPT-4.0 performance, artificial intelligence, AI in health care, medical licensing examinations, clinical decision-making

## Abstract

**Background:**

Artificial intelligence (AI) has significantly impacted health care, medicine, and radiology, offering personalized treatment plans, simplified workflows, and informed clinical decisions. ChatGPT (OpenAI), a conversational AI model, has revolutionized health care and medical education by simulating clinical scenarios and improving communication skills. However, inconsistent performance across medical licensing examinations and variability between countries and specialties highlight the need for further research on contextual factors influencing AI accuracy and exploring its potential to enhance technical proficiency and soft skills, making AI a reliable tool in patient care and medical education.

**Objective:**

This systematic review aims to evaluate and compare the accuracy and potential of ChatGPT-3.5 and 4.0 in medical licensing and in-training residency examinations across various countries and specialties.

**Methods:**

A systematic review and meta-analysis were conducted, adhering to the PRISMA (Preferred Reporting Items for Systematic Reviews and Meta-Analyses) guidelines. Data were collected from multiple reputable databases (Scopus, PubMed, JMIR Publications, Elsevier, BMJ, and Wiley Online Library), focusing on studies published from January 2023 to July 2024. Analysis specifically targeted research assessing ChatGPT’s efficacy in medical licensing exams, excluding studies not related to this focus or published in languages other than English. Ultimately, 53 studies were included, providing a robust dataset for comparing the accuracy rates of ChatGPT-3.5 and 4.0.

**Results:**

ChatGPT-4 outperformed ChatGPT-3.5 in medical licensing exams, achieving a pooled accuracy of 81.8%, compared to ChatGPT-3.5’s 60.8%. In in-training residency exams, ChatGPT-4 achieved an accuracy rate of 72.2%, compared to 57.7% for ChatGPT-3.5. The forest plot presented a risk ratio of 1.36 (95% CI 1.30-1.43), demonstrating that ChatGPT-4 was 36% more likely to provide correct answers than ChatGPT-3.5 across both medical licensing and residency exams. These results indicate that ChatGPT-4 significantly outperforms ChatGPT-3.5, but the performance advantage varies depending on the exam type. This highlights the importance of targeted improvements and further research to optimize ChatGPT-4’s performance in specific educational and clinical settings.

**Conclusions:**

ChatGPT-4.0 and 3.5 show promising results in enhancing medical education and supporting clinical decision-making, but they cannot replace the comprehensive skill set required for effective medical practice. Future research should focus on improving AI’s capabilities in interpreting complex clinical data and enhancing its reliability as an educational resource.

## Introduction

Artificial intelligence (AI) has penetrated virtually every field, ranging from telecommunications to medicine, as scientific literature has explored its potential impact, ramifications, limitations, and potential uses. In the health care industry, AI has made significant inroads: it has personalized treatment plans and simplified workflows [1]. Radiology has undergone a remarkable revolution: the US Food and Drug Administration approved AI-equipped devices in 2023 [2]. Through advanced algorithms and data processing techniques, it can aid in making informed clinical decisions [3]. Nevertheless, the complex procedure of clinical decision-making involves much more than pattern recognition and predictive analytics. However, some studies have highlighted the efficacy of artificial cognitive empathy, which highlights the growth of AI in the “soft skills” department, that is, empathy, ethics, and judgment, of clinical practice [3,4].

Perhaps AI’s biggest breakthrough was ChatGPT, launched by OpenAI in November 2022 [5]. ChatGPT is a large language model (LLM) trained on a vast dataset designed to mimic human responses. A freely available conversational AI model, ChatGPT, has revolutionized health care and medical education. Through the simulation of clinical scenarios, students were able to gain therapeutic insights. Academic evaluation is another widely explored use. Some work has shown its potential in improving communication skills. The latest version of OpenAI’s LLM has also allowed for the customization of teaching and learning plans by tailoring to individual preferences [6].

Despite the significant advancements in medical education and patient care, the performance of ChatGPT in special medical licensing examinations (MLEs) has been inconsistent, varying between countries and among specialties. One of the studies demonstrated that GPT showed the highest accuracy in Italian examinations (73% correct answers) and the lowest in French examinations (22% correct answers) [1]. Another study found that the LLM could not pass the National Specialty Examination in the Polish Education System. On the contrary, GPT-3 demonstrated tremendous performance in the United States Medical Licensing Examination (USMLE). These highlight the variance and disparity of ChatGPT’s accuracy across different exams, raising questions about its reliability in particular contexts and the general structure of the examinations [7].

There is also much interest regarding the accuracy gap between ChatGPT-3.5 and ChatGPT-4. The latter version was the improved one due to its training on a larger dataset. In a study involving the USMLE, ChatGPT-4 outperformed ChatGPT, correctly answering 90% compared to ChatGPT-3.5’s 2.5% [8,9].

In light of these advancements, it is evident that AI, particularly in the form of LLMs such as ChatGPT, has significantly impacted health care, medical education, and clinical decision-making. The disparity between ChatGPT-3.5 and ChatGPT-4 performances, as well as the variability between countries and specialties, underscores the importance of refining these technologies and adapting them to specific medical systems and environments. Although systematic reviews have been done, but to our knowledge, no systematic review and meta-analysis are available so far on comparative analysis and accuracy of ChatGPT versions [10-12].

Due to the inconsistent performance of AI models across various MLEs, there is a need for further research into the contextual factors that influence AI accuracy. Moreover, as AI continues to evolve, there is a pressing need to explore its potential in enhancing not only technical proficiency but also “soft skills” such as empathy and ethical judgment, ensuring that AI becomes a reliable, holistic tool in patient care and medical education. Despite the rapid advances in AI, especially LLMs such as ChatGPT, its performance on medical licensing and in-training examinations remains inconsistent across countries and specialties. This study set out to systematically evaluate and compare the accuracy of ChatGPT versions 3.5 and 4.0 when tested on real medical licensing and residency exams from around the world. Our research specifically asked: Does GPT-4 offer a meaningful improvement in answering medical exam questions compared to GPT-3.5? To answer this, we conducted a systematic review and meta-analysis of studies published between January 2023 and July 2024, following the PRISMA (Preferred Reporting Items for Systematic Reviews and Meta-Analyses) guidelines. We expected, based on prior evidence, that GPT-4 would outperform GPT-3.5, given its expanded training and improved reasoning capabilities. The findings are intended to guide medical educators, exam boards, and AI developers in understanding the strengths and limitations of these tools, and how they might fit into medical training and assessment in the years ahead.

## Methods

The review followed the PRISMA guidelines for conducting systematic reviews and meta-analyses.

### Information Sources

Data were sourced from reputable databases such as Scopus, PubMed, Wiley Online Library, JMIR Publications, Wolters Kluwer OVID-SP, Hindawi, Taylor & Francis, Science Direct, ProQuest, Sage Publications, BMJ, and Google Scholar in July 2024.

### Search String

We systematically reviewed the related literature using different sets of keywords, such as “ChatGPT” OR “medical license exam” OR “ChatGPT” OR “medical education” OR “ChatGPT” OR “USMLE” OR “AI in medical education” OR “artificial intelligence” OR “ChatGPT” OR “LLM” OR “exam performance” OR “machine learning” OR “ChatGPT accuracy” OR “Generative Pre-trained Transformer” OR “accuracy in medical exam” OR “license exam” OR “healthcare exam” OR “clinical exam” OR “ChatGPT-3.5 and 4 in residency exam” OR “accuracy of ChatGPT” OR “ChatGPT and authenticity” AND “Generative Model” (Survey* OR qualitative* OR editorial* OR questionnaire* OR letter to editor* OR empirical study).

### Inclusion Criteria

This review includes studies focused on the application of ChatGPT in medical education and its involvement in medical licensing exams, such as the USMLE and in-training residency programs, published between January 2023 and July 2024. Only English-language studies were considered to ensure clarity and accessibility. The studies included range from peer-reviewed journal articles, editorials, and case reports to letters to the editor, conference papers, meeting papers, and dissertations. By incorporating a variety of study types, the review aims to provide a comprehensive understanding of the role of ChatGPT-3.5 and 4.0 in medical education, capturing detailed research findings, expert perspectives, real-world case examples, emerging trends, and original research.

### Exclusion Criteria

Studies that did not meet the specific focus of this review were excluded, including book chapters, as they tend to provide broad overviews rather than original research focused on ChatGPT’s application in medical education. Additionally, any studies on ChatGPT’s use in fields outside of medical education, such as higher education disciplines (eg, engineering and humanities) or unrelated health care domains, were excluded to maintain a sharp focus on the intersection of AI and medical education.

### Study Selection and Data Extraction

The PRISMA diagram outlines the process of selecting 53 studies for inclusion. Each study was analyzed using a material extraction framework, which included author and publication year, title, country, methodology, key findings of ChatGPT-3.5 and 4.0, advantages and disadvantages, conclusions, and type of exam.

To ensure the reliability of the screening and eliminate duplicates, 3 independent reviewers (TRM, SA, and SMKA) reviewed abstracts. Any discrepancies between the 3 were reconciled by senior reviewers (UA and GF). Abstracts were downloaded and screened using the Rayyan.ai tool, with csv file formats. The selected manuscripts were also screened independently for full text by the authors (AJ and RMHS), and disagreements were resolved by discussion with the lead reviewer (MZB).

Authors (SA, TRM, UA, and GF) independently extracted and synthesized the comparative accuracy data from the included studies in the Rayyan.ai tool. These were done manually; no automation tools were used. Any discrepancies in extracted data were discussed and resolved by consensus with reviewers (AJ and MZB). Data extraction included sensitivity, accuracy, precision, and CIs. A meta-analysis of aggregated data was conducted with a random-effects inverse-variance model, with RevMan 5.4.

The PRISMA chart outlines the systematic study selection process for evaluating ChatGPT in MLEs. Initially, 1215 records were identified from multiple databases, including PubMed, Scopus, and Google Scholar. After removing 732 duplicate records, 496 studies underwent title and abstract screening, resulting in 79 relevant records. Of these, 75 full-text articles were assessed for eligibility, with 4 excluded due to unavailability. Nine additional articles were excluded for irrelevance, leaving 66 studies for the final review. The primary reasons for exclusion during screening included non-English language and studies unrelated to MLEs (eg, knowledge tests or library use). This rigorous process ensured a focused and comprehensive analysis of ChatGPT’s performance in medical exams.

### Quality Assessment

**Figure 1:**
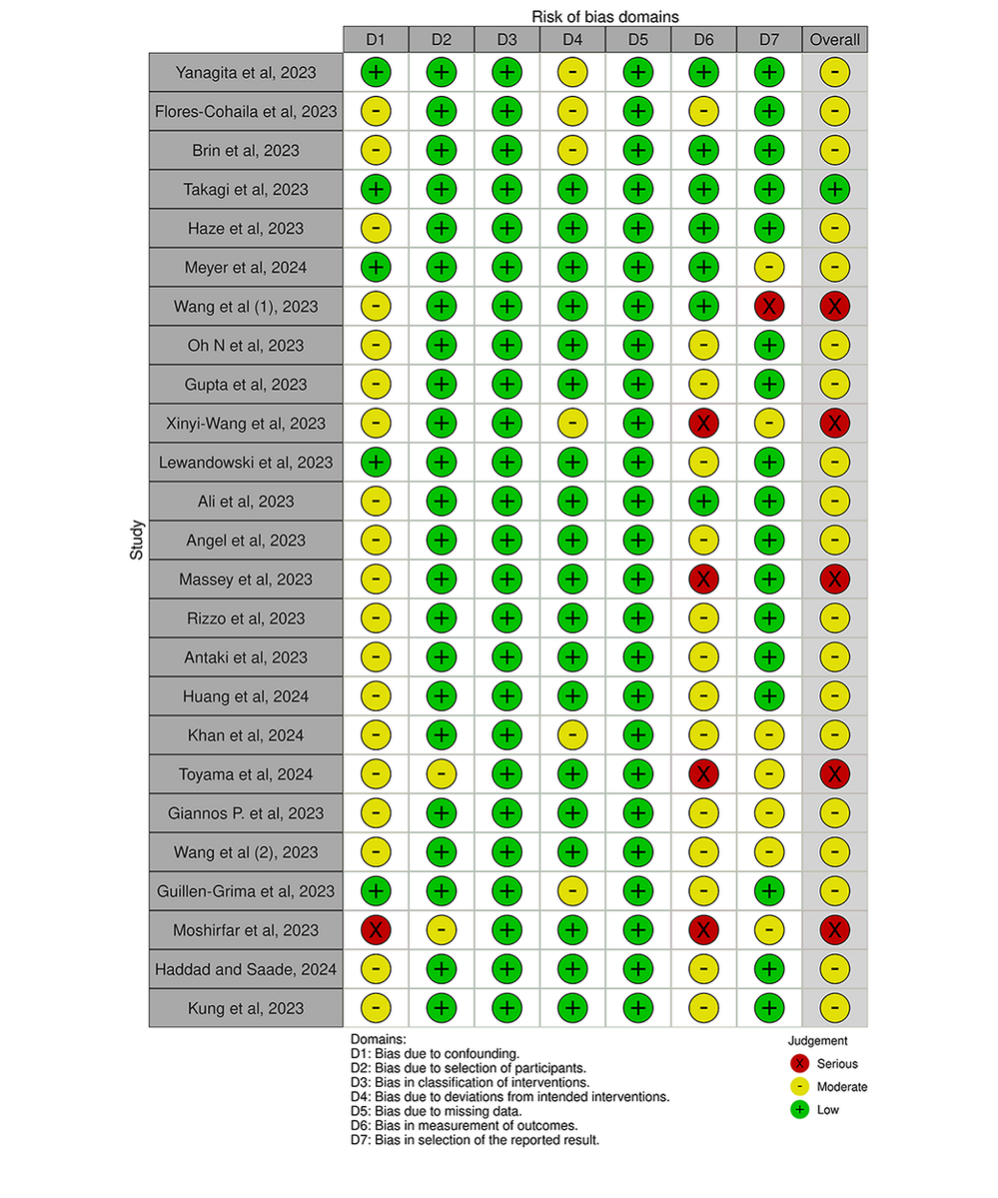
Risk of bias assessment [13-17,19,20,28,31,32,35,40-52].

The Cochrane Collaboration tool was used to assess Risk of Bias in Nonrandomized Interventional Studies (ROBINS-I) by using the following domains (Figure 1): (1) bias due to confounding, (2) bias due to selection of participants, (3) bias in classification of interventions, (4) bias due to deviations from intended interventions, (5) bias due to missing data, (6) bias in measurement of outcome, and (7) bias in selection of the reported results. According to predefined criteria 2, the domains were rated as “low risk,” “unclear risk,” and “high risk.” The risk in all domains was low across studies included in the comparative meta-analysis, including Flores-Cohaila et al [13], Takagi et al [14], and Lewandowski et al [15], and indicates good methodological quality and bias. A frequent finding in studies by Brin et al [16] and Meyer et al [17] is a moderate risk of confounding bias (D1) denoted by yellow symbols in this domain across multiple studies. This means that confounding factors were not entirely isolated, which possibly might affect the study outcomes [16,17].

Studies presenting a serious risk of bias in one or more domains, such as Wang et al [46], were noted. In particular, any studies in which substantial bias was identified in either outcome measurement or in the selection of reported results (ie, domains D6 and D7) were marked with a red indicator. Examples include Angel et al [19], Moshirfar et al [43], and Toyama et al [45]. The serious risks substantially undermine the internal validity of the study findings and need to be duly considered in the context of the conclusions. Though many of the included studies were rated as having a low to moderate risk of bias, few general methodological flaws and a few critical concerns regarding confounding control and outcome reporting were identified. The strong accent on the importance of bias minimization, rigorous study design, and transparent reporting highlights the need for meticulous attention to detail to enhance the validity of evidence collated in systematic reviews and meta-analyses.

## Results

### Comparison of the Accuracy of ChatGPT Versions in Medical Licensing and Training Residency Examinations

**Figure 2:**
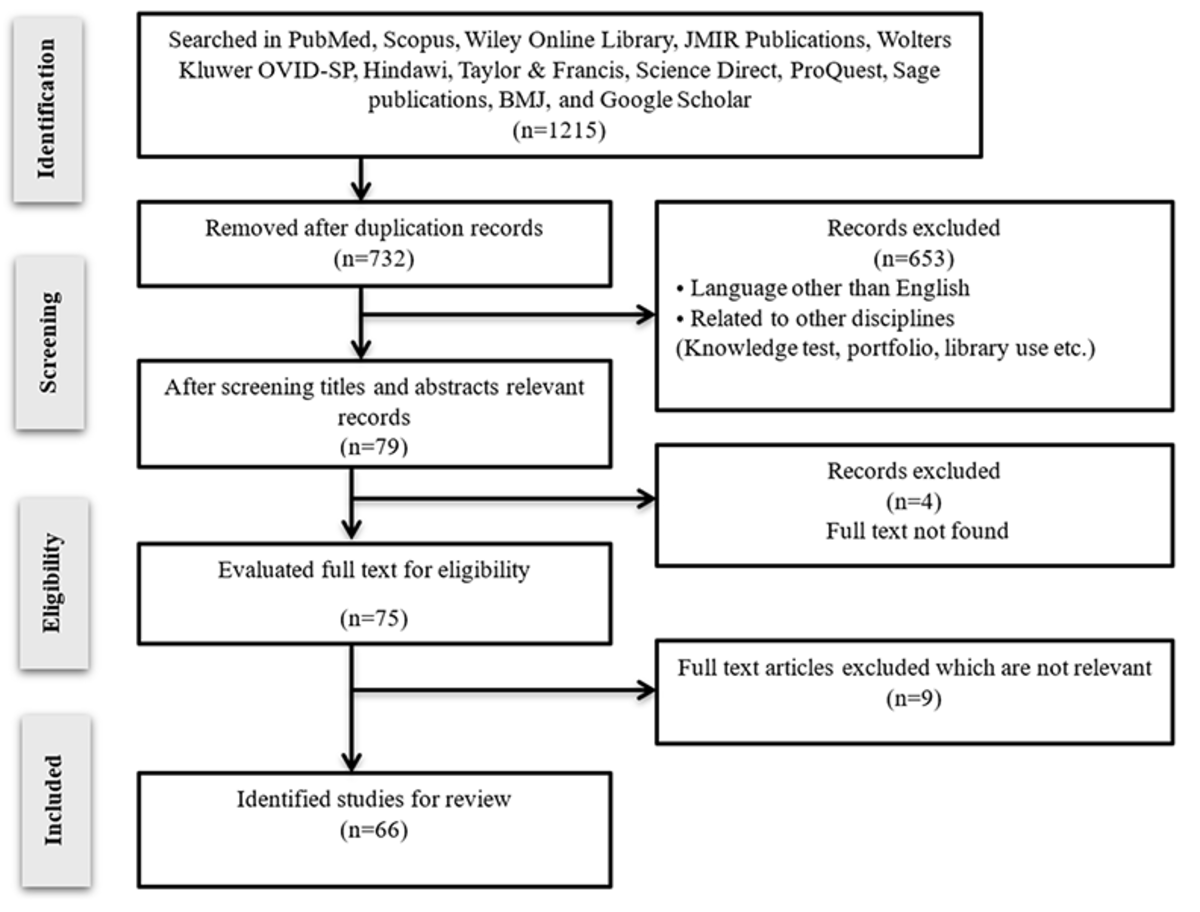
PRISMA (Preferred Reporting Items for Systematic Reviews and Meta-Analyses) flowchart of study selection process for ChatGPT in medical licensing examinations.

Figure 2 presents the PRISMA flowchart illustrating the study selection process for ChatGPT in medical licensing and residency examinations.

Table 1 shows 53 studies carried out by several researchers showing the capability of ChatGPT-3.5 and ChatGPT-4 in medical licensing and in-training residency exams conducted in various countries around the globe. It also shows the comparative evaluation of the success rate of both versions of ChatGPT in licensing examinations. Advantages and disadvantages are also described in this table.

**Table 1:** Overview of studies comparing the accuracy of ChatGPT-3.5 and 4.0 in medical licensing and in-training residency exams.

Study ID	Exam type	Dataset and methods		ChatGPT-4.0 accuracy		ChatGPT-3.5 accuracy	Advantages	Disadvantages
Aljindan et al (2023) [18]	Saudi Medical Licensure Exam	A dataset of 220 questions across 4 medical disciplines.		88.6%		None	Useful supplementary educational tool.	A high margin of error (4-16%) emphasizes that it should be used with traditional study methodology.
Angel et al (2023) [19]	American Board of Anesthesiology (ABA) Exam	The basic exam used the full set of 60 MCQs^a^ on the ABA website. The advanced exam was formulated using the book *Anesthesia Review: 1000 Questions and Answers to Blast the BASICS and Ace the ADVANCED* by randomly choosing 5/70 questions.		GPT-4 scored 47/60 (78.33%) in the basic exam. In the advanced exam, the model scored 80%.		GPT-3 scored 35/60 (58.33%).	The performance of the AI^b^ in this process was impressive enough that it leads one to question whether we should not already, as highly trained clinicians, be using these AI systems to help us avoid some of the more common cognitive errors that may occur during critical events.	The models struggle to fully comprehend questions involving numerical calculations, leading to imprecise responses. Additionally, numerical calculations demand greater precision and exactness, which may not always be within the model’s capabilities.
Antaki et al (2023) [20]	Ophthalmology	Two 260-question simulated exams from the Basic and Clinical Science Course (BCSC) Self-Assessment Program and the Ophthalmology Questions online question bank were solved by ChatGPT-3.5.		None		ChatGPT Plus's accuracy increased to 59.4. Accuracy improved with easier questions when controlling for the examination section and cognitive level.	Generally, we found that ChatGPT Plus provided highly consistent and repeatable results, but some variations occurred.	ChatGPT does not have the capability to process images. This is a significant limitation because ophthalmology is a field that heavily relies on visual examination and imaging to diagnose, treat, and monitor patients.
Bartoli et al (2024) [21]	Neurosurgical residents' written exam	51 questions (open-ended and MCQ) were included in the written exam, 46 questions were generated by humans (senior staff members), and 4 were generated by ChatGPT.		None		ChatGPT scored among the lowest ranks (9/11) among all the participants. ChatGPT answered correctly to all its self-generated questions.	Different ways to exploit AI for medical education and exams are imaginable; that is, a question may be posed by a human, an answer is generated by AI, and a critical appraisal is then elaborated by the human, based on their own logical thinking, knowledge, and personal experience and creativity.	At this stage, it seemed like AI did not “intend” or “think” to probe someone’s specific knowledge on a topic when generating a question, like humans do.
Beaulieu-Jones et al (2024) [22]	Surgical knowledge assessments	Two surgical knowledge assessments, the Surgical Council on Resident Education (SCORE; 167 questions) and Data-B (112 questions), were solved by ChatGPT-4.0.		ChatGPT correctly answered 71% and 68% of multiple-choice SCORE and Data-B questions, respectively.		None	The study highlights the accuracy of ChatGPT within a highly specific and sophisticated field without specific training or fine-tuning in the domain.	The findings also underscore some of the current limitations of AI, including variable performance on the same task and unpredictable gaps in the model’s capabilities.
Benirschke et al (2024) [23]	Questions from My Pathologist	61 general knowledge pathology questions were solved by ChatGPT-4.0.		98% of responses were “completely/mostly accurate,” and 82% of responses had “all relevant information.”		None	Answers to general pathology inquiries were accurate and complete, and LLMs^c^ can potentially be used to save time for pathology professionals.	Differences were seen in the completeness of the answer, with clinical pathology answers judged as more complete than anatomic pathology answers.
Brin et al (2023) [16]	USMLE^d^	80 USMLE-style questions involving soft skills, from the AMBOSS question bank. ChatGPT-3.5 and 4.0 took the examination, and the results were compared.		90%		62.5%	LLMs demonstrate impressive results in questions that test soft skills required from physicians. GPT-4 surpassed the human performance benchmark.	Models operate based on calculated probabilities for an output, rather than human-like confidence.
Chen et al (2023) [24]	Neurology written board examination questions	509 eligible questions out of the 560 from BoardVitals QBank (without images) were solved by ChatGPT-4.0.		335/509 questions (65.8%) on the first attempt and 383/509 (75.3%) in the subsequent iterations.		None	ChatGPT was sensitive to questions regarding depression and suicide and referred us to a suicide hotline.	The base model lacks visual input and thus is unable to process image-based questions. There were differences in the accuracy of different subject fields.
Cheung et al (2023) [25]	A multinational prospective study	50 MCQs were generated by ChatGPT-4.0 with two standard undergraduate medical textbooks. Another 50 MCQs were drafted by 2 professors using the same.		None		The total time required for ChatGPT to create the 50 questions was 20 minutes and 25 seconds.	As demonstrated in our study, a reasonable MCQ can be written by ChatGPT using simple commands with a text reference provided.	Including ChatGPT, most AI models are trained by the vast content available on the internet, and their reliability and credibility are questionable. Moreover, many AI models were found to have significant bias due to their training data.
Ebrahimian et al (2023) [26]	Iranian Medical Licensing Examination	200 MCQs were translated into English. The accuracy of performance by ChatGPT-4.0 was assessed.		68.5% (surpassed the passing criteria of 45%)		None	ChatGPT is capable of answering MCQs, surpassing the pass mark by a significant margin.	The expertise and nuanced understanding of medical cases that human physicians possess are not yet matched by AI models.
Fang et al (2023) [27]	Chinese National Medical Licensing Examination	Out of 600 questions, 340 were common questions, and 260 case analysis questions were solved by ChatGPT-4.0.		442/600 (73.67%), surpassing the passing criteria, that is, 360.		None	ChatGPT exhibits a high level of answer-explanation concordance in the Chinese language.	ChatGPT’s performance declines when handling encoded questions, declining by 40.5%, 9.7%, 32.4%, and 41.8% for Units 1, 2, 3, and 4, respectively.
Flores-Cohaila et al (2023) [13]	Peruvian National Licensing Medical Examination (ENAM)	180 multiple-choice questions were performed by ChatGPT-3.5 and 4.0, and the results were compared.		86% (155/180). GPT-4 surpassed almost 90% of examinees.		77% (139/180), with prompting. GPT-3.5 surpassed 80% of examinees.	Role-play and context-setting in prompts improved performance, reducing incorrect answers. Outperformed human performance.	LLM expertise is limited to passing a licensing exam. To be a practitioner requires communication skills, empathy, and so on.
Giannos, (2023) [28]	UK Neurology Specialty Certificate Examination	69 questions from the Pool—Specialty Certificate Examination (SCE) Neurology Web Questions bank were solved by both versions of ChatGPT.		ChatGPT-4.0 achieved the highest accuracy of 64%, surpassing the passing threshold and outperforming its predecessors across disciplines and subtopics.		ChatGPT-3.5 displayed an overall accuracy of 57%.	ChatGPT-4 has shown promise in attaining specialty-level medical knowledge. This sets a new benchmark for AI models in specialized medical education and practice.	ChatGPT3’s performance in the specialized field of neurology and neuroscience is lower than that in general medical examinations. Specialty examinations require a deeper understanding of specific medical domains.
Gilson et al (2023) [29]	USMLE	4 datasets with 389 questions were solved by ChatGPT-3 and compared with medical students’ performance in steps 1 and 2.		None		AMBOSS Step 1: 44% (44/100); AMBOSS Step 2: 42% (42/100); NBME^e^ Step 1: 64.4% (56/87); NBME Step 2: 57.8% (59/102)	The model may facilitate the creation of an on-demand, interactive learning environment for students. Logical explanations for answers are always provided, even if incorrect.	Incorrect answers are related to logical and information-based errors mostly.
Gobira et al (2023) [30]	Brazilian National Examination for Medical Degree Revalidation	81 nonnullified and 14 nullified questions. ChatGPT-4.0 solved questions in various specialties.		For nonnullified questions, 71/81 (87.7%); for nullified questions, 71.4% (10/14).		None	There was no statistically significant difference in the performance across different specialties.	Encountered challenges when tackling questions that involved concepts related to the Brazilian public health care system and ethical decisions.
Guillen-Grima et al (2023) [31]	Spanish MIR (Medical Resident Intern)	182 questions were solved by both versions of ChatGPT and compared.		Spanish: 86.81% (81.13-90.98); English: 87.91% (82.38-91.88).		Spanish: 63.18% (55.98-69.85); English: 66.48% (59.35-72.94)	Improved consistency demonstrated by GPT-4 across multiple attempts presents the refinements in training and better underlying model architecture.	The error rate of 13.2% is of concern. 25 questions linked to an image and 3 challenge questions were excluded.
Haddad et al (2024) [32]	Ophthalmology Examinations	Questions from the USMLE Step 1 (n=44), Step 2 (n=60), and Step 3 3 (n=28) were extracted from AMBOSS, and 248 questions were extracted from the book *Ophthalmology Q&A Board Review* and solved by both versions, and results were compared for accuracy.		GPT-4.0 achieved a total of 70% of correct answers. GPT-4.0 answered 70.45% of questions correctly in step 1, 90.32% in step 2, 96.43% in step 3, and 62.90% in the remaining 248 questions.		GPT-3.5 achieved a total of 55% of correct answers. GPT-3.5 answered 75% of questions correctly in step 1, 73.33% in step 2, 60.71% in step 3, and 46.77% in the OB-WQE^f^.	ChatGPT can be a great add-on to mainstream resources to study for board examinations. There have been reports of using it to generate clinical vignettes and board examination–like questions.	Many questions were excluded due to them containing images, which is a considerable limitation considering the visual nature of ophthalmology.
Hoch et al (2023) [33]	otolaryngology subspecialties	A dataset covering 15 otolaryngology subspecialties was collected from an online learning platform funded by the German Society of Oto-Rhino-Laryngology, Head and Neck Surgery, and was given to ChatGPT-3.5 for solving.		None		The dataset included 2576 questions (479 multiple-choice and 2097 single-choice), of which 57% (n=1475) were answered correctly by ChatGPT.	As an educational resource, the performance of ChatGPT indicated potential efficacy in offering educational assistance in specific subspecialties and question formats.	ChatGPT delivered a considerable number of incorrect responses within specific otolaryngology subdomains, rendering it unreliable as the sole resource for residents preparing for the otolaryngology board examination.
Huang et al (2024) [34]	SPTEMD^g^ Stage 1	600 MCQs extracted from 3 separate tests conducted in February 2022, July 2022, and February 2023 were solved by ChatGPT-4.0.		525/600 (87.5%)		None	Potential to facilitate not only the preparation for exams but also improve the accessibility of medical education and support continuous education for medical professionals.	The model performed inconsistently across different subjects, exhibiting comparatively lower performance in anatomy, parasitology, and embryology.
Huang et al (2023) [35]	University of Toronto Family Medicine Residency Progress Test	The 108 questions were stratified into 11 areas of family medicine knowledge and solved by both versions of ChatGPT.		82.4% (89/108). It scored much higher than an average resident, that is, 56.9%.		57.4% (62/108). Its performance was comparable to the average resident, that is, 56.9%.	GPT-4 demonstrates a broad knowledge base and strong reasoning abilities in FM^h^, as evidenced by its high level of accuracy and logical justification.	Hallucinations raise concerns about a model’s integrity and overall accuracy, and may mislead learners into believing an incorrect response to be correct.
Jain et al (2023) [36]	Orthopedic In-Training Examination (OITE)	Of 635 questions, 360 were usable as inputs (56.7%) by ChatGPT-3.5.		None		ChatGPT-3.5 scored 55.8%, 47.7%, and 54% for the years 2020, 2021, and 2022, respectively.	The potential of machine learning in medicine is that it can automate tasks, assist in providing thought processes, and improve management.	Tendency to fabricate references or have incorrect reasoning when solving problems that require logic beyond this date.
Jang et al (2023) [37]	K-NLEKMD^i^	340 (114 questions for recall, 99 for diagnosis, and 127 for intervention) were solved by ChatGPT-4.0 and assessed.		66.18% (after model optimization)		None	We found that high consistency of response is associated with increased accuracy for questions.	Models had weak performance on questions that require understanding the Korean language and TKM^j^/Korea-adapted health care. LLMs are susceptible to hallucinations.
Knoedler et al (2023) [38]	USMLE	2069 USMLE Step 3 practice questions. 1840 entered into GPT-3.5, while a subset of 229 entered into GPT-4, and performance was compared.		84.7% (194/229)		56.9% (1047/1840)	ChatGPT-4 showcases its superiority as a newer model, with better accuracy.	ChatGPT-4 encountered difficulties in questions related to cardiology and neurology.
Kufel et al (2023) [39]	Polish specialty exam in radiology	This study used a PES^k^ exam consisting of 120 questions, provided by the Medical Examinations Center in Lodz. The performance of ChatGPT-4.0 was assessed.		None		ChatGPT did not reach the pass rate threshold of the PES exam (52%).	We identified that questions for which ChatGPT provided a correct answer had a significantly higher confidence index. Therefore, the confidence index can be considered a parameter indicating a higher likelihood of ChatGPT providing a correct answer.	The performance of the ChatGPT model in passing the specialist in radiology and imaging diagnostics examination in Poland remains uncertain.
Kung et al (2023) [41]	Orthopedic In-Training Examination (OITE)	OITE 2020, 2021, and 2022 questions without images were input into ChatGPT version 3.5 and version 4.0 with zero prompting.		GPT-4 answered 265 of 360 questions correctly, corresponding to the average performance of a PGY-5^l^.		ChatGPT answered 196 of 360 answers correctly (54.3%), corresponding to a PGY-1 level.	Multiple studies have suggested that deep learning models for automated image analysis can be synergistic with clinicians, resulting in superior predictions compared with those of clinicians alone.	Finally, in medicine specifically, there can be multiple potentially correct answers to a given question with only one best answer, which may cause difficulty for the AI when there is correct information supporting each answer.
Lewandowski et al (2023) [15]	Specialty Certificate Examination in Dermatology	Three Specialty Certificate Examination in Dermatology tests, in English and Polish, consisting of 120 single-best-answer, multiple-choice questions each, were solved by both versions of AI and compared.		The percentages of correct answers to questions in the Polish vs English versions obtained by ChatGPT-4.0 were 77.3% vs 84%, 75.8% vs 85% and 71.4% vs 80.7%, respectively.		The percentages of correct answers to questions in the Polish vs English versions obtained by ChatGPT-3.5 were 61.3% vs 68.9%, 60.8% vs 70%, and 54.6% vs 60.5%, respectively.	It is estimated that ChatGPT is capable of understanding and communicating in more than 100 languages at various levels. ChatGPT-4.0 proved to be highly effective in answering clinical picture-type questions.	An important limitation of ChatGPT and the other language models is a rare phenomenon of artificial hallucination, defined as self-conscious, seemingly realistic responses by an AI. We did not observe it in this study, but it is widely described in the literature.
Lin et al (2024) [53]	Taiwan Medical Licensing Examination	80 single-choice questions were assessed by ChatGPT-4.0.		Accuracy in medical exams ranged from 63.75% to 93.75%. The highest accuracy was in the February 2022 exam.		None	The implementation of the “Chain of Thought” prompt strategy proved effective, enabling the model to correct its initial incorrect responses and achieve an accuracy rate exceeding 90%.	Falters in questions related to surgical precautions and decision-making. It had a 100% failure rate in such questions. Overall, it is not ready for complex decision-making.
Long et al (2024) [54]	Otolaryngology Head and Neck Surgery Certification Examinations (OHNS)	21 open-ended questions were adopted from the Royal College of Physicians and Surgeons of Canada’s sample examination. The accuracy of ChatGPT-4.0 was assessed on them.		Average of 75% across 3 trials in the attempts and demonstrated higher accuracy with prompts.		None	Information provided by the AI was clinically valid; it could be used to provide equitable access to resources in low-resource settings where access to such information may not be readily available.	Hallucinations may present benign or harmful misinformation, with significant implications in the field of medicine.
Lum (2023) [55]	Orthopedic In-Training Examination (OITE)	Random selection of 400/3840 publicly available questions based on the Orthopedic In-Training Examination, and compared the mean score with that of residents.		None		ChatGPT selected the correct answer 47%	AI can handle large amounts of data that can be quickly accessed. The LLM performed better at recognition and recall-type questions, as well as comprehension and interpretation, than at problem-solving and application of knowledge.	The model may have limitations in terms of its ability to integrate, synthesize, generalize, and apply factual knowledge in more nuanced ways.
Mackey, et al (2024) [56]	USMLE	Questions from AMBOSS were used. 900 questions from 9 specialties, that is, 100 questions from each specialty, were randomly selected. Performance by ChatGPT-4.0 was assessed for accuracy.		89% of the total questions were answered accurately.		None	ChatGPT-4.0 had an impressive performance in areas like psychiatry, neurology, obstetrics, and gynecology.	LLMs have specialty-specific strengths and weaknesses. Its accuracy was notably lower in pediatrics, emergency medicine, and family medicine.
Mahajan et al (2023) [57]	Otolaryngology Residency In-Service Exam	1088 questions from the BoardVitals otolaryngology bank were solved by ChatGPT-3.5 and accuracy was assessed.		None		572/1088 (53%) yielded a correct answer, and 586/1088 (54%) yielded a correct explanation.	LLM can accurately answer complex multiple‐choice patient care questions to an extent.	The accuracy rate is far below an acceptable level for it to be useful in a clinical or educational setting to aid in decision-making.
Maitland et al (2024) [58]	MRCP^m^ (UK)	Practice questions for MRCP 1 and 2 produced by MRCPUK were solved by ChatGPT-4.0 (images excluded).		For part 1, ChatGPT provided 170 accurate responses for 196 questions. In part 2, it provided responses for 127/128 questions correctly.		None	ChatGPT is able to answer MRCP written examination questions, without additional prompts, to a level that would equate with a comfortable pass for a human candidate.	LLMs are known to “hallucinate” plausible sounding, but false, facts, information, and even references.
Massey et al (2023) [42]	Orthopedic Assessment Examination	180 questions and answer choices from 9 different orthopedic subspecialties (excluding images). The accuracy of both versions was compared.		GPT-4 scored 47.2% with a 95% CI of 40%-54.5%. The average response time was 31.8 seconds.		GPT-3.5 scored 29.4% with a 95% CI of 23.2%-36.4%. The average response time for ChatGPT-3.5 was 12.1 seconds.	The implications and potential of ChatGPT are still quite promising. GPT-4 showed particular improvement when answering questions requiring no image interpretation.	Chatbots cannot decipher radiographic images in conjunction with clinical vignettes.
Meyer et al (2024) [17]	German Medical Licensing Examination	937 original MCQs from German medical licensing examinations were solved by ChatGPT-3.5 and 4.0, and the accuracy of both was compared.		796/937 (85%)		548/937 (58%)	There is potential for using GPT-3.5 and GPT-4.0 in a medical education tutoring environment.	The model's inconsistencies across different specialties restrain recommendations for its use by the general population for medical purposes.
Morjaria et al (2023) [59]	Undergraduate Medical Program	40 problems used in prior assessments were used: 30 submitted to ChatGPT-3.5. For the remaining 10 problems, we retrieved past minimally passing student responses.		None		ChatGPT-generated responses received a mean score of 3.29 out of 5, compared to 2.38 for a group of students meeting minimum passing marks, presenting higher performance.	This represents success on a different form of assessment compared to the multiple-choice format. ChatGPT's performance was shown to be comparable to students who are performing at the minimum standard.	ChatGPT's performance was shown to be comparable to students who are performing at the minimum standard on these assessments, but slightly lower when compared to a historical student cohort.
Moshirfar et al (2023) [43]	StatPearls ophthalmology questions	467 questions from the StatPearls Question Bank were solved by both versions of ChatGPT and compared.		73.2%		55.5%	GPT-4.0 outperformed humans who had an accuracy of 58.3%.	The “lens and cataract” category presented a unique challenge for the model.
Nakao et al (2024) [60]	Japanese National Medical Licensing Examination	108 questions that had 1 or more images as part of a question were solved by ChatGPT-4.0.		68% (73/108) when presented with images and 72% (78/108) when presented without images.		None	For the clinical questions, for which sufficient information was available in the text form, GPT-4V was able to choose the correct answers solely from the textual information (76/98, 78%).	GPT-4V cannot effectively interpret images related to medicine.
Oh et al (2023) [44]	Korean general surgery board exams	The dataset comprised 280 questions from the Korean general surgery board exams conducted between 2020 and 2022 and solved by both versions of ChatGPT.		GPT-4 demonstrated a significant improvement with an overall accuracy of 76.4%.		GPT-3.5 achieved an overall accuracy of 46.8%.	Active surgeons who completed their training over a decade ago may find LLMs helpful for continuous medical education, especially as a supplementary resource.	Instead of providing strictly accurate information, they generate responses based on the probability of the most appropriate words given the data they have been trained on.
Panthier et al (2023) [61]	European Board of Ophthalmology examination	GPT-4 was exposed to a series of EBO^n^ examination questions in French, covering various aspects of ophthalmology, and performance was compared to experts.		ChatGPT correctly answered 6188 out of 6785 questions, demonstrating a high level of competency in ophthalmology.		None	ChatGPT’s proficiency in clinical management and decision-making suggests that it could be a valuable resource for practicing ophthalmologists and other medical professionals seeking information and guidance on complex cases.	ChatGPT was unable to interpret figures, graphs, or tables. In addition, some epidemiological data were unavailable online, and some standards of medical care had recently changed; thus, for certain questions, ChatGPT could not give the correct answer.
Riedel et al (2023) [62]	German OB/GYN^o^ exams	The dataset of questions from the OB/GYN course included 160 questions, and the dataset of the medical state exam included 104 questions that were solved by an AI machine.		None		ChatGPT provided correct answers 85.6% of the time. In the second round, ChatGPT achieved similarly good results for the dataset, with 88.7% of answers being correct from the OB/GYN course, and 70.4% correct in the Medical State Exam.	ChatGPT can perform intricate tasks related to handling complex medical and clinical information in the field of OB/GYN. ChatGPT provided consistent answers and explanations to medical problems and did not require help in finding the correct solutions.	Finding raises doubts about ChatGPT’s adaptability to varying levels of difficulty and emphasizes the need both for further fine-tuning and for incorporating data. Another potential limitation is the inability of ChatGPT to process images.
Saad et al (2023) [63]	FRCS^p^ Orthopedic Exam	Questions were sourced from a bank of FRCS (Orth) Part A mock questions compiled from various resources. We divided the questions into two mock examinations.		ChatGPT-4.0 achieved an overall score of 67.5% on Part A. For component one of the Part A exams, ChatGPT-4.0 scored 60/120. For the second component of Part A, ChatGPT-4.0 scored 102/120 (85%).		None	Its relatively stronger performance in anatomy-based and shorter questions may be attributed to its ability to recall and provide information from its training data.	The inability of ChatGPT-4.0 to effectively handle complex clinical scenarios and image-based questions suggests limitations in its understanding and interpretation of intricate medical information.
Sarangi et al (2024) [64]	Radiology Case Vignettes (FRCR2A examination)	120 MCQs were solved by both ChatGPT-3.5 and residents, and the results were compared.		None		45%	In some of the cases, AI could point out the answers, but residents could not.	AI models currently lack the accuracy levels of human professionals, as the residents outscored them (63.33% and 57.5%). AI gave an inaccurate explanation in 50% of the cases.
Scaioli et al (2023) [65]	Italian Medical Residency Exam	136 questions classified into clinical cases and notional questions. These were solved by ChatGPT-3.0, and the accuracy was determined.		None		90.44%, with higher performance on clinical cases (92.45%) than on notional questions (89.15%).	ChatGPT's performance was higher than 99.6% of the participants. Potential for being a tool for learning.	Overreliance on these tools may develop, leading to a decline in the physician’s adaptive judgment.
Skalidis et al (2023) [66]	European Exam in Core Cardiology (EECC)	After filtering, 362 MCQ items (ESC^q^ sample: 68; BHDRA^r^: 150; and StudyPRN^s^: 144) were included to be solved by ChatGPT-3.5.		None		ChatGPT answered 340 questions out of 362, with 22 indeterminate answers in total, and an overall accuracy of 58.8%.	ChatGPT correctly answered the majority of questions and showed consistency across all different MCQ sources, exceeding 60% in most analyses. It exceeds the passing mark.	ChatGPT is designed for natural language processing tasks and thus currently only accepts text-based inputs, resulting in the exclusion of all questions with image content.
Surapaneni (2023) [67]	Medical biochemistry	The performance of ChatGPT was evaluated in medical biochemistry using 10 randomly selected clinical case vignettes.		None		ChatGPT generated correct answers for 4 questions on the first attempt.	Large language models such as ChatGPT may enhance student engagement and learning by assisting in web-based learning by generating pertinent and comprehensive content.	According to the findings of our study, ChatGPT may not be considered an accurate information provider for application in medical education to improve learning and assessment.
Takagi et al (2023) [14]	Japanese Medical Licensing Examination (JMLE)	254 MCQs on essential knowledge and clinical skills were performed by ChatGPT-3.5 and 4.0 and compared (questions containing tables, images, and underlining were excluded).		79.9% overall, with 87.2% in essential knowledge, 73.3% in general clinical, and 81.7% in specific disease.		50.8% overall, with 55.1% in essential knowledge, 43.8% in general clinical, and 56.3% in specific diseases.	GPT-4 satisfied the JMLE passing criteria unlike GPT-3, showing better proficiency in Japanese.	Concerns related to hallucinations.
Toyama et al (2024) [45]	JRBE^t^	103 questions from JRBE 2022 were used. These questions were categorized by pattern, required level of thinking, and topic.		GPT-4 scored 65% (GPT-4 correctly answered 93.3% of the questions in nuclear medicine (n=15). In questions on radiological general knowledge, it scored 90%.		ChatGPT scored 40.8% (42/103). In the questions in nuclear medicine (n=15), ChatGPT scored 40% (*P*=.01). In questions on radiological general knowledge, it scored 30%.	GPT-4 passed the JRBE with an overall score of 65%.	They behaved confidently, although their responses differed from their previous choices for the same question. Such unfavorable responses, entirely grounded in incorrect evidence or factual inaccuracies, are commonly labeled as “hallucinations.”
Wang et al (2023) [46]	Chinese National Medical Licensing Examination (NMLE) 2021 and 2022	4 units with 150 questions per unit were solved by ChatGPT-3.5. The performance of ChatGPT-3.5 was compared with medical students.		None		275/600 (45.8%) in the 2021 NMLE and 219/600 (36.5%) in the 2022 NMLE.	ChatGPT holds the possibility of serving as a virtual medical mentor.	ChatGPT’s proficiency in questions pertaining to the Chinese NMLE is not yet at par with Chinese medical students.
Wang et al (2024) [47]	Pathology Domain-Specific Knowledge	Google Forms were sent out to 15 participants, who each asked 1 short-answer question from both versions of ChatGPT.		Met or exceeded expectations in 12/15 of the questions.		Met or exceeded expectations in 9/15 of the questions.	Can provide quick and easily accessible information about various pathology topics, with newer LLMs also having the capability to provide references for the data used in the response.	Answers included unnecessarily lengthy responses containing irrelevant material, awkwardness of language, and provision of incorrect information.
Watari et al (2023) [68]	GM-ITE^u^ examination	The GM-ITE examination had 137 questions for the years 2020, 2021, and 2022 to conduct a comparative analysis with only single-choice answers, excluding audio and visual cues. These were solved by ChatGPT-4.0.		GPT-4 scores were significantly higher than the mean scores of residents.		None	GPT-4 demonstrated remarkable proficiency in the detailed disease knowledge section, which requires an in-depth understanding of diseases, as well as in more challenging questions and domains.	GPT-4 seemed to struggle with questions in the “medical interview and professionalism” and “psychiatry” categories, which are typically easier for residents.
Wójcik et al (2024) [69]	Polish medical specialization licensing exam (PES)	120 questions were solved by ChatGPT-4.0.		80/120 (67.1%)		None	ChatGPT may be used as an assistance tool in medical education.	Mastery over exam content based mostly on rote learning does not translate to the practice of medicine, which is fundamentally based on human interactions.
Yu et al (2024) [70]	Chinese Postgraduate Examination for Clinical Medicine	A dataset of 165 medical questions that were divided into three categories—(1) common questions, (2) case analysis questions, and (3) multiple-choice questions—was solved by ChatGPT-3.5.		None		ChatGPT scored 153.5 out of 300 for original questions in Chinese. However, ChatGPT had low accuracy in answering open-ended medical questions, with only 31.5% total accuracy.	ChatGPT demonstrated a high level of internal concordance, which suggests that the explanations provided by ChatGPT support and affirm the given answers. Moreover, ChatGPT generated multiple insights toward the same questions.	Poorer accuracy was linked to lower concordance and a lack of insight. Thus, it was hypothesized that inaccurate responses were primarily driven by missing information, which could result in reduced insight and indecision in the AI.
Zong et al (2024) [71]	Chinese NMLE^v^ (2017-2021)	3000 questions across 5 exams were solved by ChatGPT-3.5.		None		Average accuracy of 53.05%.	ChatGPT excelled in questions related to clinical epidemiology, human parasitology, and dermatology. No significant difference in performance on case-based and non-case-based questions.	ChatGPT has limited training in languages like Chinese. It failed to meet the minimal benchmark of 60%.
Sahin et al (2024) [72]	Turkish Neurosurgical Society Proficiency Board Exams (TNSPBE)		100 questions from the last 6 TNSPBE were used, 77 with visual elements were excluded, leaving 523 for analysis.		79% (412 out of 523 questions answered correctly).	None	ChatGPT can quickly provide accurate explanations and supplementary learning material, making it a valuable tool for exam preparation.	ChatGPT may generate incorrect or misleading answers with convincing explanations, which can misguide candidates if not verified.
Yanagita et al (2023) [40]	National Medical Licensing Examination in Japan		All 400 questions from the 2022 NMLE in Japan were included; 292 targeted questions (after exclusion) were analyzed.		81.5% (237 out of 292 questions answered correctly).	42.8% (125 out of 292 questions answered correctly).	GPT-4 reached passing standard; performance could improve as the model relearns.	GPT is limited to written questions.
Guerra et al (2023) [73]	Congress of Neurological Surgeons Self-Assessment Neurosurgery Exam (SANS)		591 out of 643 board-style questions were included after exclusion of questions with no text.		76.6% accuracy for all questions, but 79% for text-only questions.	None	GPT-4’s accuracy suggests applications in educational settings and clinical decision-making.	GPT-4 may have potential limitations without manual input for answer suggestions.
Isleem et al (2023) [74]	Orthopaedic In-Training Examination (OITE) developed by the American Academy of Orthopaedic Surgeons (AAOS)		301 self-assessment examination questions from AAOS were included.		None	60.8% (183 out of 301 questions answered correctly).	ChatGPT has the potential to provide orthopedic educators and trainees with accurate clinical conclusions for board exam questions.	Since GPT’s reasoning needs to be carefully analyzed for clinical accuracy and validity, its usefulness in clinical educational contexts is limited.
Gupta et al (2023) [48]	Plastic Surgery Inservice Training Examination (PSITE)		250 sample assessment questions from the 2022 PSITE were obtained from the American Council of Academic Plastic Surgeons website; 242 were answered by GPT.		77.3% (187 out of 242 questions answered correctly).	None	GPT-4 has shown to be more accurate and reliable for plastic surgery resident education when compared to GPT-3.5 and should be used to enhance educational curriculum.	GPT-4 may rely on inaccurate sources and may misunderstand prompts.
Wang et al (2013) [75]	Chinese (CNMLE) and English (ENMLE) datasets of the China National Medical Licensing Examination		A total of 220 questions were extracted from the SMLE^w^ test bank across 4 medical disciplines.		GPT-4 open-domain hallucinations accounted for 56% (9/16), 43% (6/14), and 83% (15/18), while close-domain hallucinations accounted for 44% (7/16), 57% (8/14), and 17% (3/18), respectively.	GPT-3.5 scored 56%, 76%, and 62% on CNMLE, ENMLE, and NEEPM, compared to GPT-4’s 84%, 86%, and 82%. Verbal fluency exceeded 95% across responses.	GPT-4 is a highly valuable AI-assisted tool in medical education.	GPT-4 had difficulty in answering difficult and complex questions.
Ali et al (2023) [49]	Self-Assessment Neurosurgery Examination Indications Examination		149 questions from the Self-Assessment Neurosurgery Examination Indications Examination were used to query LLM accuracy.		Overall accuracy of 82.6%.	Overall accuracy of 62.4%.	Potential value and applications of LLMs such as GPT-4 in neurosurgical education and in clinical decision-making.	The use of multiple-choice questions to quantify LLM knowledge for higher-order neurosurgical topics incompletely captures the open-ended nature of the true neurosurgery oral board examination.
Gravina et al (2024) [76]	Italian National Residency Admission Exam (SSM23)		Multiple-choice gastroenterology-focused questions were chosen from the 140 questions in the 2023 Italian medical specialization exam.		None	Overall accuracy of 94.11%.	ChatGPT-3.5 exhibits promise in addressing gastroenterological queries, emphasizing potential educational roles.	ChatGPT-3.5 shows variable performance, mandating cautious use alongside other educational tools.
D’Souza et al (2023) [77]	Clinical Vignettes in Psychiatry		ChatGPT-3.5’s responses to 100 clinical vignettes representing 100 different psychiatric illnesses were assessed by expert psychiatrists.		None	Grade A: 61/100 cases; Grade B: 31/100 cases; Grade C: 8/100 cases.	ChatGPT-3.5 has appreciable knowledge and interpretation skills in psychiatry.	Depending upon the query and information provided, ChatGPT-3.5 can provide varying responses.
Khan et al (2024) [50]	Anesthesiology Board-Style Examination Questions for the American Board of Anesthesiology (ABA)		A total of 884 multiple-choice questions were used from *Anesthesia: A Comprehensive Review* (6th edition), a question bank largely regarded as one of the premium study sources for ABA certification and recertification examinations.		Overall accuracy of 69.4%.	Overall accuracy of 47.9%.	GPT-4 significantly outperformed GPT-3.5.	Although GPT-4 shows promise, current LLMs are not sufficiently advanced to answer anesthesiology board examination questions with passing success.
Haze et al (2023) [51]	Japanese National Medical Examination		Questions available from three editions of the Japanese National Medical Examination administered in 2020, 2021, and 2022 were used; questions with images or those officially identified as inappropriate were excluded.		Accuracy rate of 81% and a consistency rate of 88.8%.	Accuracy rate of 56.4% and a consistency rate of 56.5%.	GPT-4 showed significantly higher percentages of correct and consistent responses than GPT-3.5.	Neither LLM reached the level required in real clinical practice.
Rizzo et al (2024) [52]	Orthopaedic In-Service Training Exams (OITEs)		All questions from the 2020–2022 Orthopaedic In-Service Training Exams (OITEs) were given to OpenAI’s GPT-3.5 Turbo and GPT-4 LLMs.		2022: 67.63%; 2021: 58.69%; 2020: 59.53%.	2022: 50.24%; 2021: 47.42%; 2020: 46.51%.	GPT-4’s performance is comparable to a second- to third-year resident, and GPT-3.5 Turbo’s performance is comparable to a first-year resident.	The application of current LLMs can neither pass the OITE nor substitute orthopaedic training.
Mannam et al (2023) [78]	Congress of Neurological Surgeons (CNS) Self-Assessment Neurosurgery (SANS) Exam Board Review Prep Questions		Questions were obtained from the Congress of Neurological Surgeons (CNS) Self-Assessment Neurosurgery (SANS) Exam Board Review Prep; the ChatGPT Output Precision Ladde was developed to evaluate the quality and accuracy of the ChatGPT output.		None	ChatGPT achieved spot-on accuracy for 66.7% of prompted questions, 59.4% of unprompted questions, and 63.9% of unprompted questions with a leading phrase. In comparison to SANS explanations, ChatGPT output was considered better in 19.1% of questions, equal in 51.6%, and worse in 29.3%.	The authors envision a future where LLMs like ChatGPT are integrated into medical education, providing explanations based on the RIME (Reporter, Interpreter, Manager, and Educator) framework.	The language and phrasing of the pain questions may not have been optimally suited for the data ChatGPT was trained on.

^a^MCQs: multiple-choice questions.

^b^AI: artificial intelligence.

^c^LLMs: large language models.

^d^USMLE: United States Medical Licensing Examination.

^e^NBME: National Board of Medical Examiners.

^f^OB-WQE: Ophthalmology Board Written Qualifying Exam.

^g^SPTEMD: Senior Professional and Technical Examinations for Medical Doctors.

^h^FM: Family Medicine.

^i^K-NLEKMD: Korean National Licensing Examination for Korean Medicine Doctors.

^j^TKM: Traditional Korean Medicine.

^k^PES: Państwowy Egzamin Specjalizacyjny (English: Polish Medical Specialization Licensing Exam).

^l^PGY-5: postgraduate year 5.

^m^MRCP: Membership of the Royal Colleges of Physicians.

^n^EBO: European Board of Ophthalmology.

^o^OB/GYN: obstetrics and gynecology.

^p^FRCS: Fellowship of the Royal Colleges of Surgeons.

^q^ESC: European Society of Cardiology.

^r^BHDRA: Braunwald’s Heart Disease Review and Assessment.

^s^StudyPRN: Study Professional Resource Network.

^t^JRBE: Japan Radiology Board Examination.

^u^GM-ITE: General Medicine In-Training Examination.

^v^NMLE: National Medical Licensing Examination in Japan.

^w^SMLE: Saudi Medical Licensing Exam.

### ChatGPT-4 Performance in Medical Licensing Exams

**Figure 3:**
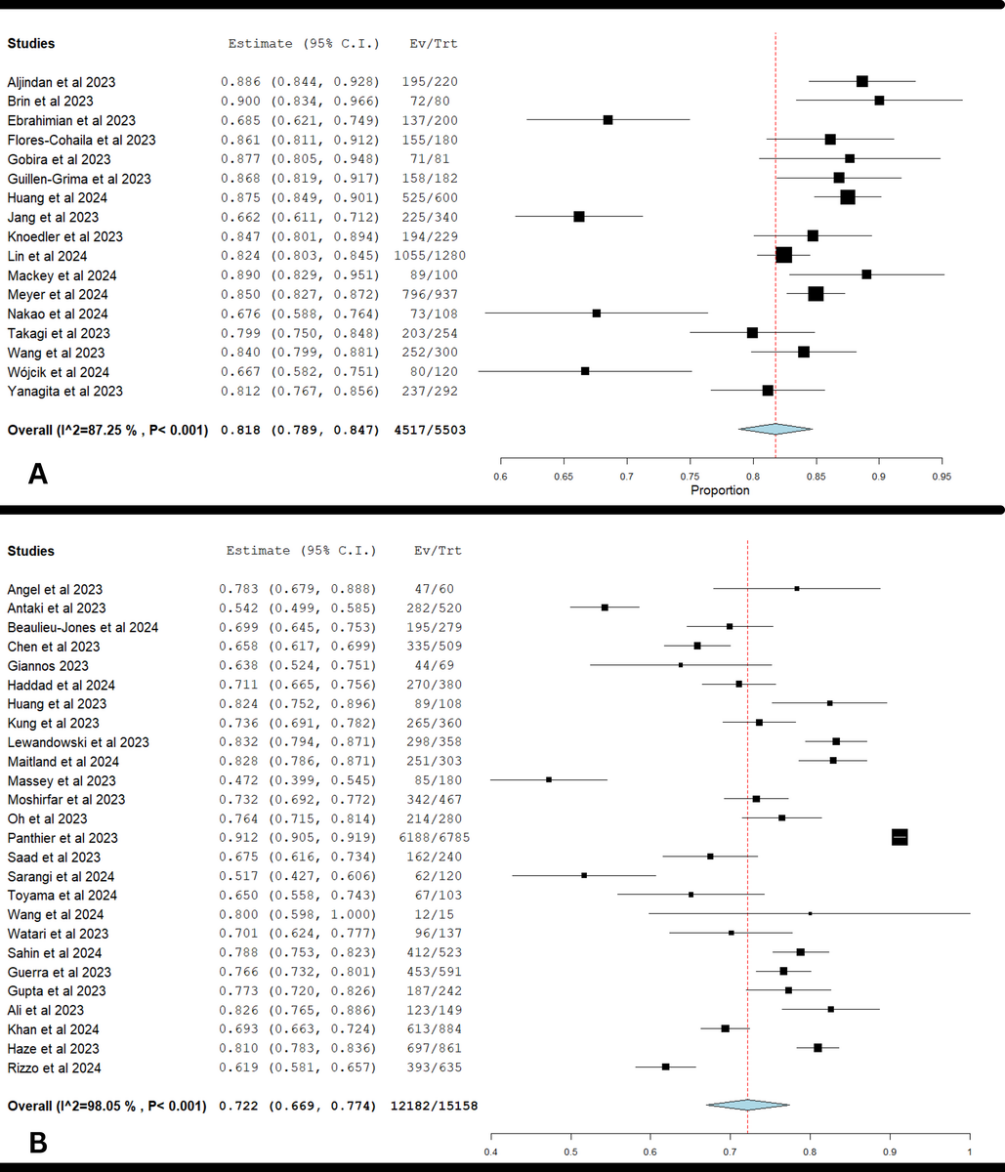
Performance of ChaGPT-4.0 in (A) medical licensing and (B) in-training residency exams [13-20,22,24,26,28,30-32,35,37,38,40-42,44-53,56,58,60,61,63,64,68,69,72,73].

The forest plot shown in Figure 3A indicated that the subject had a pooled accuracy proportion of 0.818 (95% CI 0.789-0.847) in medical licensing exams. This means that, on average, the subject correctly answered about 81.8% of the questions. However, there was significant heterogeneity among the studies, with *I*^2^=87.25% and *χ*^2^_16_=125.54 (*P*<.001). This high variability suggests that differences in study design, population, and exam content significantly influenced the accuracy estimates. Despite this variability, the subject’s high accuracy indicates its potential utility.

### ChatGPT-4 Performance in In-Training Residency Exams

The forest plot shown in Figure 3B indicated that the subject had a pooled accuracy proportion of 0.722 (95% CI 0.669-0.774) in in-training residency exams. This means that, on average, the subject correctly answered about 72.2% of the questions. However, there was significant heterogeneity among the studies, with *I*^2^=98.05% and *χ*^2^_25_=1280.36 (*P*<.001). This high variability suggests that differences in study design, population, and exam content significantly influenced the accuracy estimates. Despite this variability, the subject’s high accuracy indicates its potential utility in medical education.

### ChatGPT-3.5 Performance in Medical Licensing Exams

**Figure 4:**
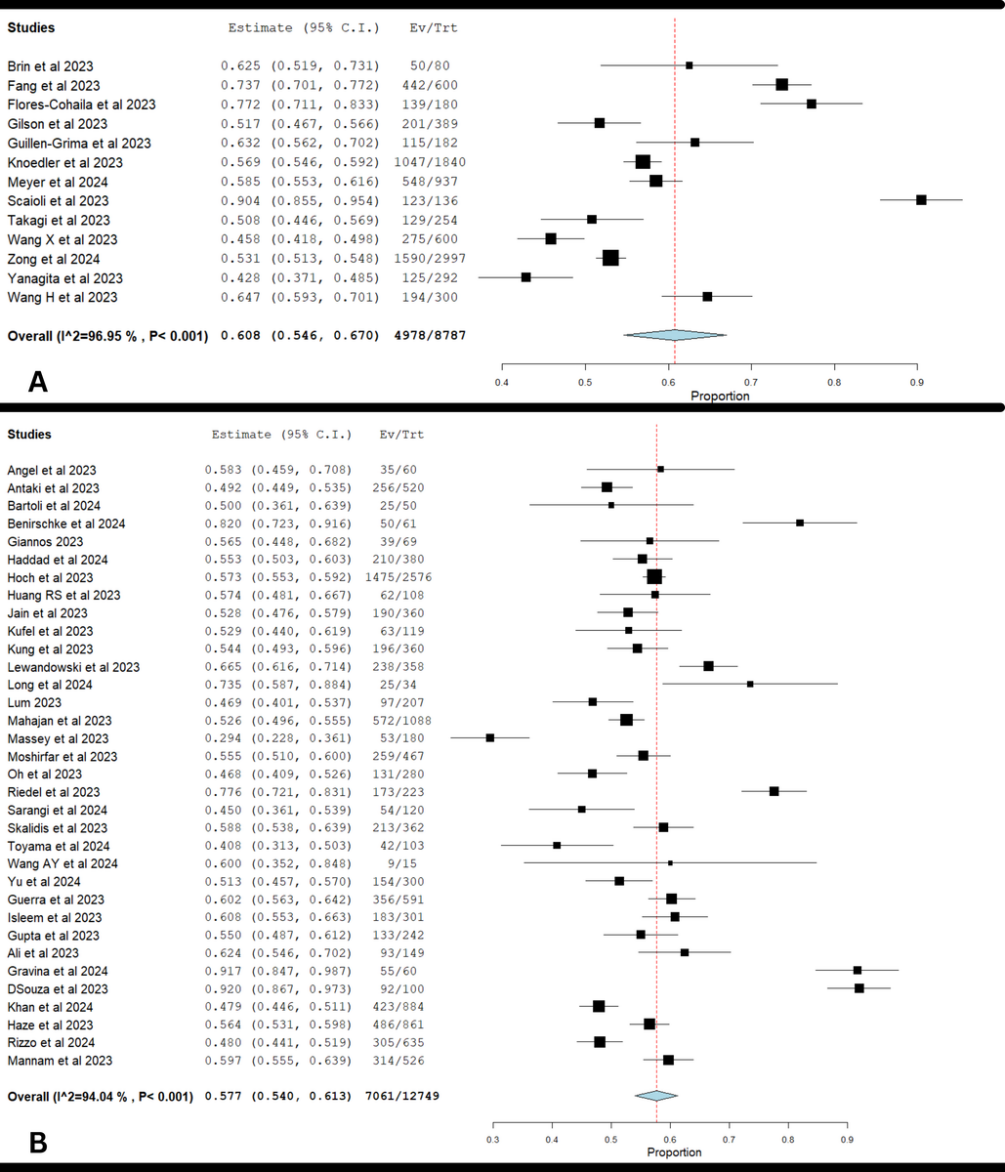
Overall performance and evaluation of ChatGPT-3.5 in (A) medical licensing exams and (B) in-training residency exams [13-17,19-21,23,27,29,31-33,35,36,38-52,54,55,57,62,64-66,70,71,73,74,77,78].

The forest plot showed a pooled accuracy proportion of 0.577 (95% CI 0.540-0.613), indicating a lower accuracy compared to ChatGPT-4 (Figure 4A). The heterogeneity was also high, with *I*^2^=94.04% and *χ*^2^_33_=554.02 (*P*<.001). This suggests considerable variability in how ChatGPT-3.5 performed across different studies. The lower accuracy and high variability underscore the need for improvements in ChatGPT-3.5’s capabilities, particularly in standardizing the conditions under which it is tested to better understand its limitations and strengths.

### ChatGPT-3.5 Performance in In-Training Residency Exams

The forest plot showed a pooled accuracy proportion of 0.608 (95% CI 0.546-0.670), indicating a lower accuracy compared to ChatGPT-4 (Figure 4B). The heterogeneity was also high, with *I*^2^=96.95% and *χ*^2^_12_=393.17 (*P*<.001). This suggests considerable variability in how ChatGPT-3.5 performed across different studies. The lower accuracy and high variability underscore the need for improvements in GPT-3.5’s capabilities, particularly in standardizing the conditions under which it is tested to better understand its limitations and strengths.

### Overall Performance of ChatGPT-4

**Figure 5:**
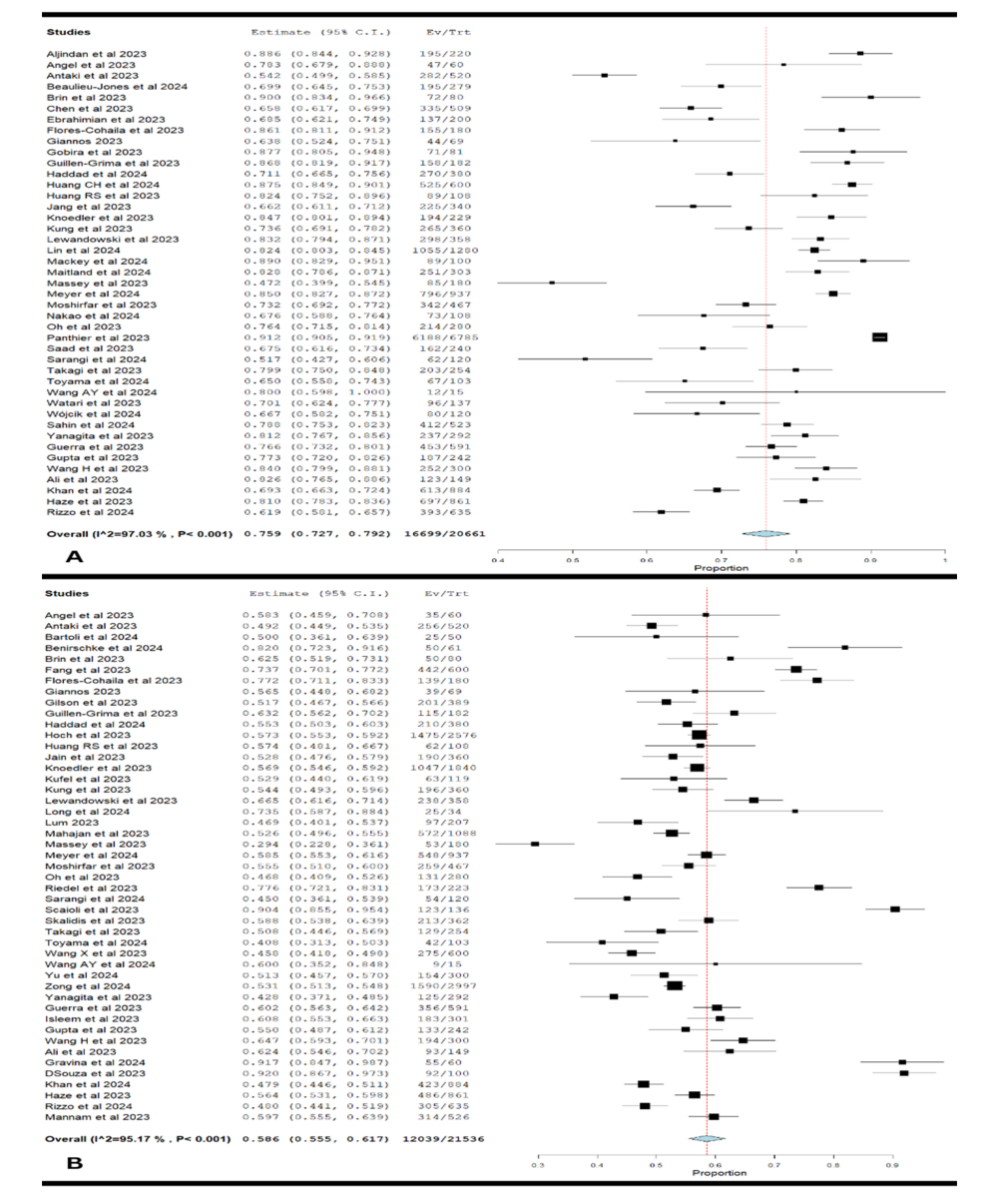
Overall performance of (A) ChatGPT-4 and (B) ChatGPT-3.5 in medical licensing exams and in-training residency exams [13-24,26-58,61-66,70,71,73,74,77,78].

The forest plot shows that ChatGPT-4 had a pooled accuracy proportion of 0.759 (95% CI 0.727-0.792) in in-training residency exams (Figure 5A). This means that, on average, GPT-4 correctly answered about 75.9% of the questions. However, there was significant heterogeneity among the studies, with *τ*=0.01, *χ*^2^_42_=1414.01 (*P*<.001), and *I*^2^=97.03%. This high variability suggests that differences in study design, population, and exam content significantly influenced the accuracy estimates. Despite this variability, ChatGPT-4’s high accuracy indicates its potential utility in medical education, though further research is necessary to understand the sources of heterogeneity and enhance its performance.

### Overall Performance of ChatGPT-3.5

The forest plot showed a pooled accuracy proportion of 0.586 (95% CI 0.555-0.617), indicating a lower accuracy compared to ChatGPT-4 (Figure 5B). The heterogeneity was also high, with *τ*=0.05, *χ*^2^_46_=951.72 (*P*<.001), and *I*^2^=95.17%. This suggests considerable variability in how ChatGPT-3.5 performed across different studies. The lower accuracy and high variability underscore the need for improvements in ChatGPT-3.5’s capabilities, particularly in standardizing the conditions under which it is tested to better understand its limitations and strengths.

### Comparative Analysis: ChatGPT-4 vs ChatGPT-3.5 in Medical Licensing and Residency Exams

**Figure 6:**
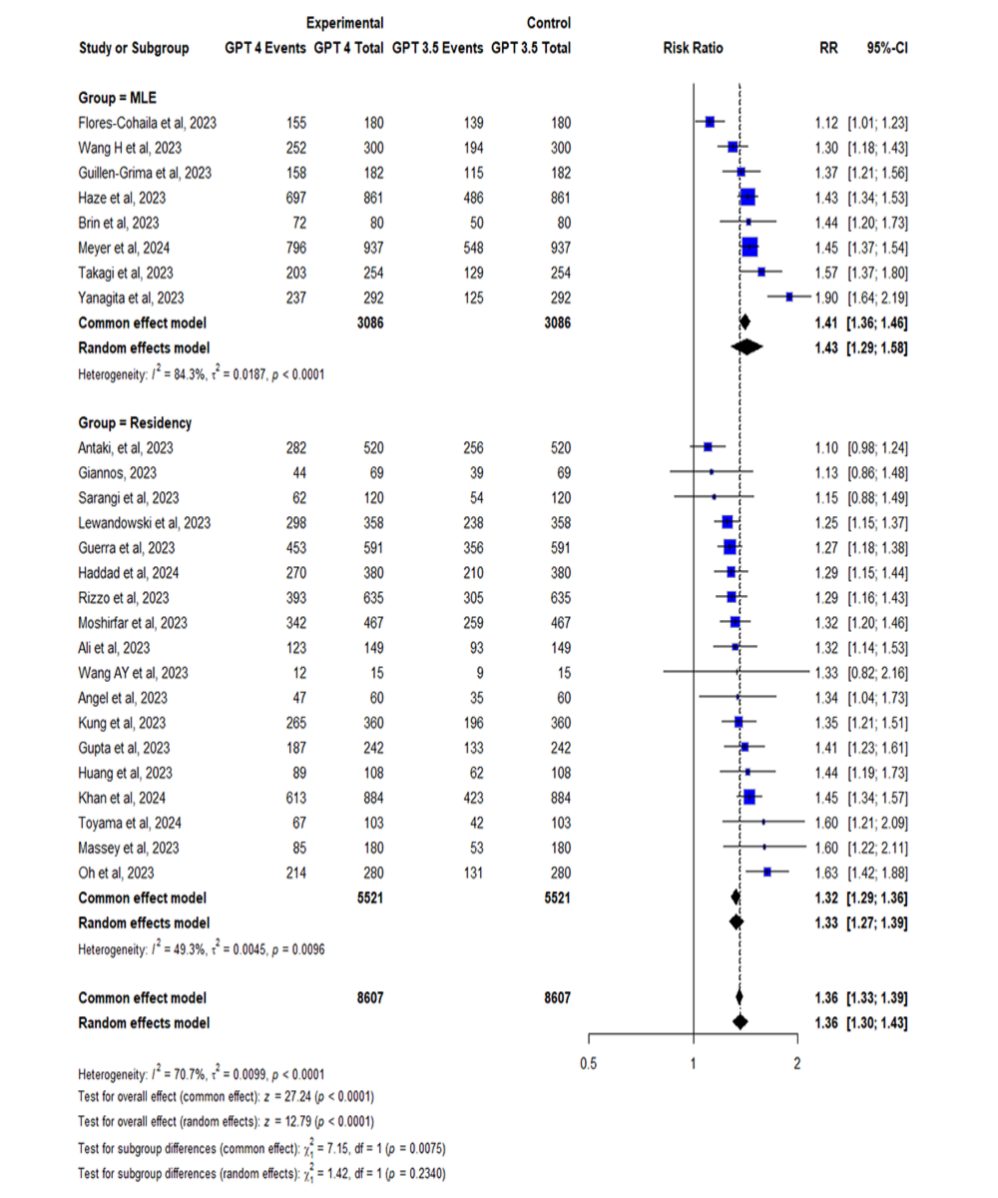
Comparative analysis: ChatGPT-4 vs ChatGPT-3.5 in medical licensing and in-training residency exams [13-17,19,20,28,31,32,35,40-45,47-52,64,73].

The comparative forest plot presented a risk ratio (RR) of 1.36 (95% CI 1.30-1.43), demonstrating that ChatGPT-4 was 36% more likely to provide correct answers than ChatGPT-3.5 across both medical licensing and residency exams, as shown in Figure 6. For medical licensing exams specifically, the RR was 1.42 (95% CI 1.30-1.56), with high heterogeneity (*I*^2^=85%). In residency examinations, the RR was 1.31 (95% CI 1.27-1.39), with moderate heterogeneity (*I*^2^=49%). These results indicate that ChatGPT-4 significantly outperforms ChatGPT-3.5, but the performance advantage varies depending on the exam type. The findings suggest that while ChatGPT-4 is generally more reliable, the context of its application (eg, type of exam) plays a critical role in its accuracy. This highlights the importance of targeted improvements and further research to optimize ChatGPT-4’s performance in specific educational and clinical settings.

**Figure 7:**
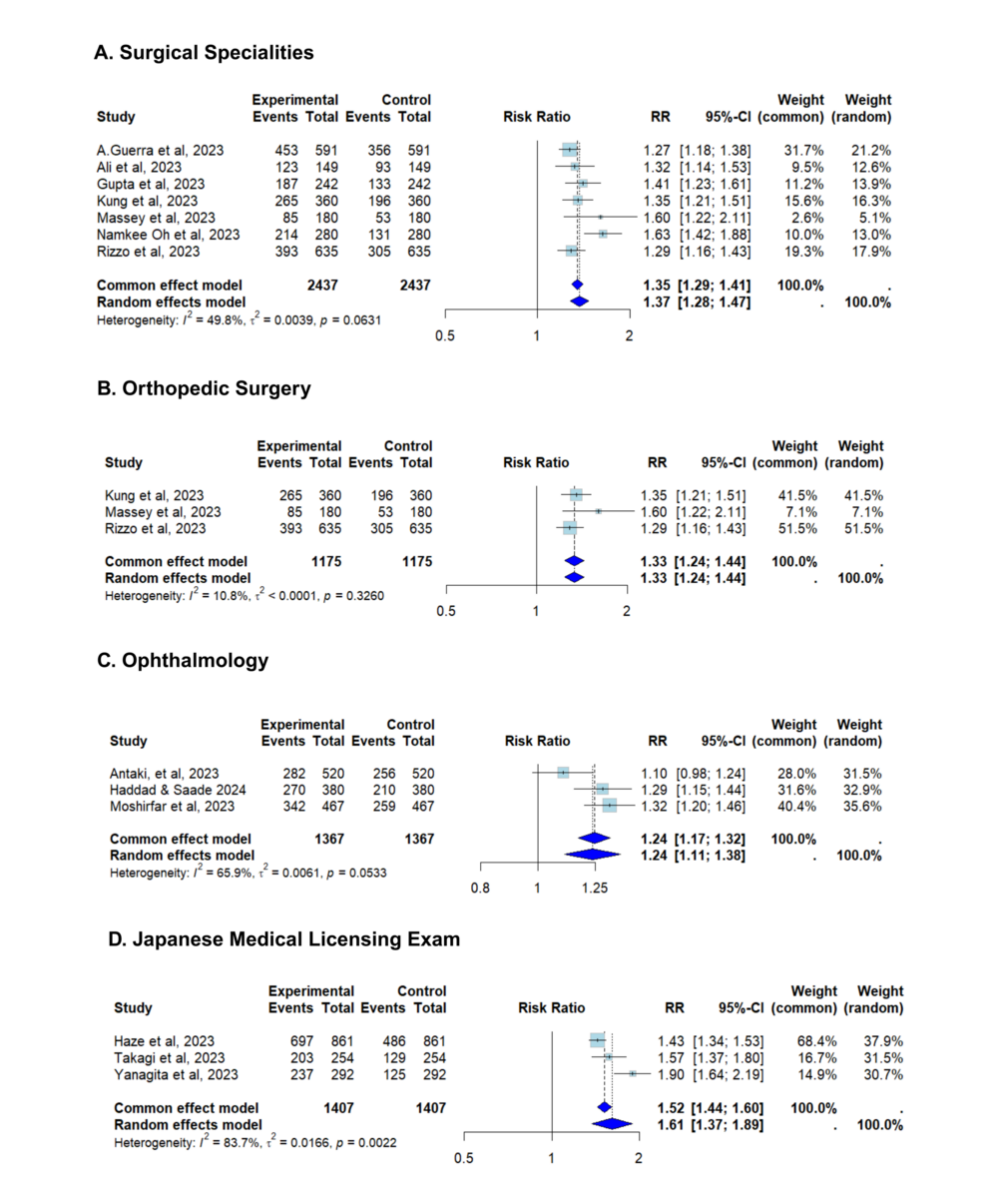
Further subgroup analyses: ChatGPT-4 vs ChatGPT-3.5 in medical licensing exams and residency exams [20,32,41-44,48,49,52,73].

Further subgroup analyses revealed distinct patterns in ChatGPT’s performance based on exam origin or medical specialty. For surgical specialties, the pooled RR was 1.37 (95% CI 1.28-1.47), with moderate heterogeneity (*I*^2^=49.8%) as shown in Figure 7. Orthopedic surgery showed similarly strong performance (RR 1.33, 95% CI 1.24-1.44), with notably low heterogeneity (*I*^2^=10.8%), indicating consistent outcomes across studies. In contrast, the ophthalmology subgroup had a lower pooled RR of 1.24 (95% CI 1.11-1.38) but higher heterogeneity (*I*^2^=65.9%), suggesting variability in ChatGPT performance possibly due to the visual or nuanced nature of the content. Performance in the Japanese Medical Licensing Exam subgroup was the most heterogeneous (RR 1.61, 95% CI 1.37-1.89; *I*^2^=83.7%), likely reflecting the linguistic and cultural specificity of the test.

The only variable studied was the examination performance of the intervention group (ChatGPT-4.0) against the comparison group (ChatGPT-3.5). For dichotomous outcomes, RRs with 95% CIs were calculated. A random-effects model was used to pool data from both the intervention group and the comparison group. The meta-analysis was performed with the Review Manager 5.4. Subgroup analyses were performed using R software (version 4.4.1; R Foundation for Statistical Computing) based on examination level, country of origin, and specialties. OpenMeta[Analyst] (Brown University) was used to check the effect sizes of each arm, including noncomparator studies, giving a more holistic review of the existing literature.

Heterogeneity was assessed using the chi-square test and quantified with the *I*^2^ statistic, with *I*^2^>50% indicating substantial heterogeneity. To explore potential sources of heterogeneity, subgroup analyses and leave-one-out sensitivity analyses were conducted. A *P* value <.05 was considered statistically significant for all analyses.

### Sensitivity Analyses and Publication Bias

**Figure 8:**
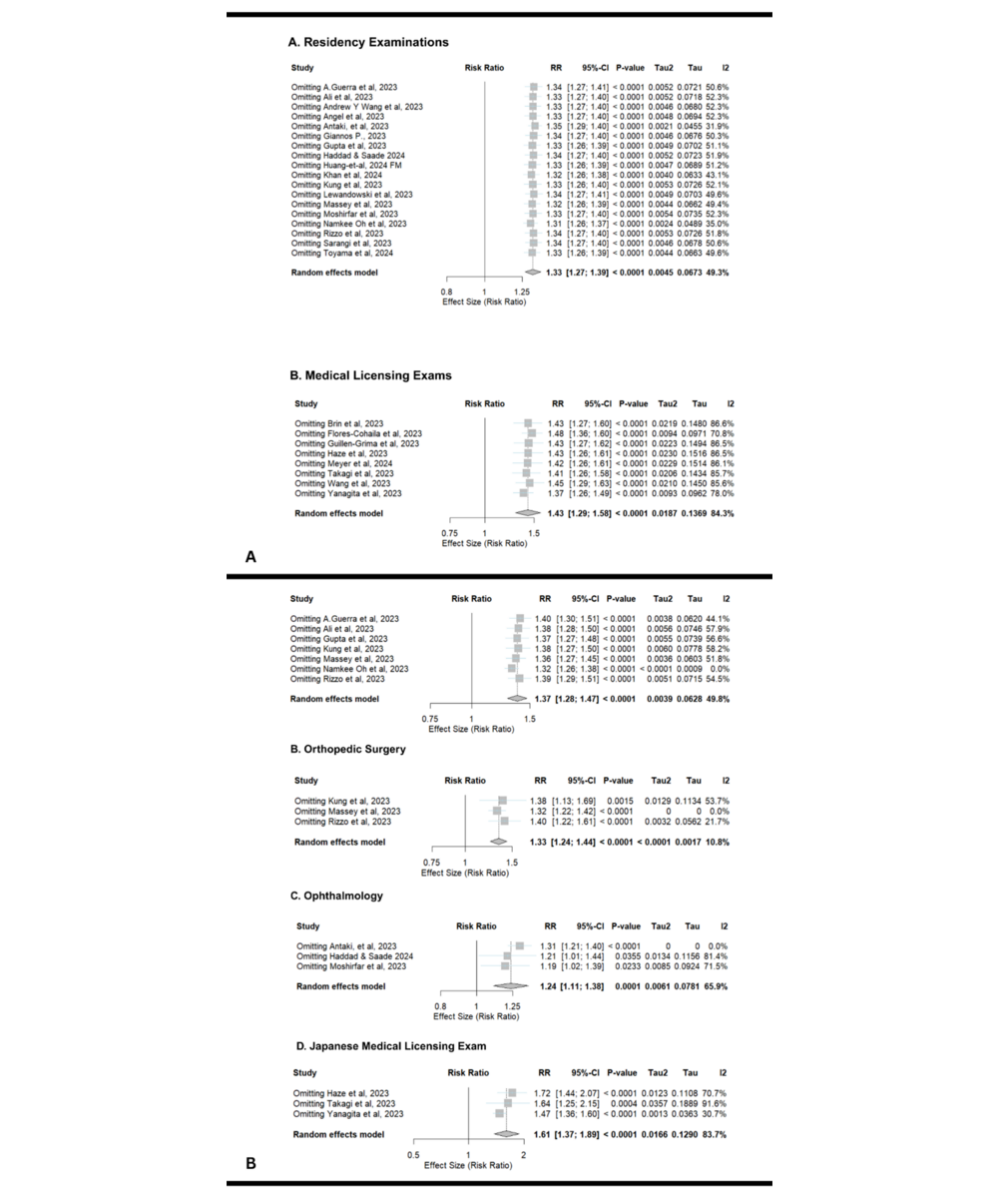
(A) Sensitivity analyses and (B) further subgroup sensitivity analyses of ChatGPT-3.5 and 4.0 in medical licensing exams and in-training residency exams [13-24,26-58,61-66,70,71,73,74,77,78].

Sensitivity analyses were conducted across key subgroups to assess the robustness of the pooled RRs and the influence of individual studies on heterogeneity (Figure 8). Among broader categories, residency exams (RR 1.33, 95% CI 1.27-1.39; *I*^2^=49.3%) exhibited consistent results across all exclusions, with negligible shifts in heterogeneity. In contrast, the medical licensing exams subgroup demonstrated persistent high heterogeneity (*I*^2^=84.3%), even after omitting studies, indicating intrinsic variability across these assessments (Figure 8). Overall, the pooled estimates proved stable across all sensitivity models. The sources of heterogeneity appeared to be distributed across multiple studies rather than driven by a single outlier, reinforcing the internal consistency and robustness of the main findings.

For surgical specialties, no single study unduly influenced the overall estimate (RR 1.37, 95% CI 1.28-1.47), with heterogeneity remaining moderate (*I*^2^=49.8%) across exclusions. Notably, omitting [44] reduced heterogeneity to 0%, while other exclusions led to only marginal reductions in *I*^2^ values. In orthopedic surgery, all leave-one-out models preserved significance (RR 1.33, 95% CI 1.24-1.44). The exclusion of [42] notably eliminated heterogeneity (*I*^2^=0%), suggesting it was a moderate contributor to between-study variance. Ophthalmology showed more sensitivity to individual studies, with RR values ranging from 1.19 to 1.31 and *I*^2^ fluctuating between 0% and 81.4%. Omitting [20] fully resolved heterogeneity (*I*^2^=0%), while the exclusion of [43] led to substantial reductions in *I*^2^, indicating a significant source of inconsistency. For the Japanese Medical Licensing Exam subgroup, heterogeneity remained high (*I*^2^=83.7%) across all models. However, the exclusion of [14] resulted in notable reductions in *I*^2^, confirming that all 3 studies contributed to the observed variability (Figure 8B).

Publication bias was evaluated using the Egger linear regression test for funnel plot (Figure 8) asymmetry. The test yielded a nonsignificant result (t_24_=0.15, 2-tailed; *P*=.88), indicating no evidence of small-study effects or significant publication bias in the included studies. These findings suggest that the funnel plot asymmetry observed is unlikely due to publication bias and may instead reflect genuine heterogeneity across studies (Multimedia Appendix 1).

## Discussion

### Performance Comparison of ChatGPT-3.5 and ChatGPT-4 in Medical Examinations

The study highlights the promising potential of AI-driven language models such as ChatGPT-3.5 and 4.0 in revolutionizing the medical field by assessing their performance across a range of medical licensing and in-training exams worldwide. The findings revealed that ChatGPT-4 consistently outperformed its predecessor, ChatGPT-3.5, in accuracy and proficiency [69,70], thanks to the more advanced training data and significant algorithmic improvements.

In this study, ChatGPT-4 demonstrated a pooled accuracy of approximately 75.9% in in-training residency exams, showcasing its strong overall performance. In comparison to ChatGPT-3.5, ChatGPT-4 was 36% more likely to provide correct answers across both medical licensing and residency exams, with an RR of 1.36 (95% CI 1.30-1.43). This suggests that ChatGPT-4’s advancements have translated into tangible improvements in accuracy, particularly in multiple-choice question formats. For medical licensing exams specifically, ChatGPT-4 had an even higher RR of 1.42 (95% CI 1.30-1.56), indicating a significant performance edge over ChatGPT-3.5 [71].

However, there was notable heterogeneity in the performance of both models, with high *I*^2^ values (85% for licensing exams and 49% for residency exams), reflecting variations in study design, population, and exam content that impacted the accuracy estimates. These findings are similar to Zong et al [71]. These findings underscore the importance of contextual factors in applying AI models like ChatGPT-4. They also highlight the need for targeted improvements and further research to enhance its performance, ensuring that it becomes a more reliable and effective tool for specific educational and clinical settings [79,80,81].

Further subgroup and sensitivity analyses provide critical insights into the generalizability and robustness of ChatGPT’s performance across the medical licensing exam. The lower heterogeneity among surgical and orthopedic specialties indicates that the ChatGPT models succeed in domains with structured procedural knowledge. These results suggest that ChatGPT may potentially serve as an adjunct learning tool in surgical education [35,45,59].

The above findings were in contrast with high heterogeneity in ophthalmology and Japanese licensing exams, which correspond to the fields with less predictable ChatGPT performance. Some potential explanations include the visual nature of ophthalmologic evaluations, discrepancies in linguistic constructs, and culturally specific clinical reasoning models. These findings highlight the critical requirement for more localized training datasets and a standardized mechanism to ensure generalizability [80]. The stability of effect estimates across sensitivity analyses reinforces the robustness of the meta-analytic findings. Small changes in pooled RRs after the removal of any individual study alleviate concerns about publication bias or undue influence from outliers [71,81].

These observations align closely with the findings of several systematic reviews and meta-analyses. Liu et al [10] conducted one of the most comprehensive meta-analyses to date, demonstrating that ChatGPT-4 generally outperforms its predecessors across a variety of standardized medical exams, although performance fluctuates based on question structure, language, and specialty content. The author highlighted that while LLMs are strong in knowledge-based tasks, their clinical reasoning capabilities are notably weaker, particularly in real-world scenarios or open-ended problem-solving formats [80].

The authors [80,81] presented important insights into the role of AI as an educational tool. Both studies emphasized the importance of viewing AI not as a replacement for human reasoning, but as a potential companion to strengthen formative assessment and feedback in medical education, provided its limitations are clearly understood and accounted for. LLMs’ medical exam accuracy at 0.61, USMLE accuracy at 0.51, and ChatGPT's higher accuracy of 0.64 support our observation of ChatGPT-4.0’s superior performance. The study also emphasized LLMs’ potential to address health care challenges but stressed the need for rigorous evaluation frameworks. Their rubric framework provides a structured approach to ensure safe and ethical integration of LLMs into clinical and educational settings, aligning with our call for standardized evaluation metrics and greater transparency in future research [80].

Beyond technical accuracy, the issue of AI’s ability to simulate human soft skills, such as empathy and ethical reasoning, has also been discussed extensively in the literature. The literature addressed the concept of artificial cognitive empathy, concluding that despite improvements in language generation, AI still lacks the depth of emotional understanding and situational awareness that human physicians develop through clinical experience [79,82]. Similarly, certain studies emphasized the need for careful integration of AI in both educational and clinical environments, cautioning that overconfidence in AI-generated answers could introduce safety risks if used without appropriate human oversight [83,84]. This is particularly relevant in high-stakes scenarios like licensing examinations, where even minor inaccuracies can have significant consequences. In addition, Artsi et al [11] highlighted the risk of “overreliance” on AI models during the learning process, which could unintentionally affect the development of independent critical thinking among medical students, an observation that complements the variability we observed in AI performance across exam types and specialties [11].

In our study, the performance of AI models in medical licensing exams varies widely across different countries. For example, while ChatGPT-3.5 performed relatively well in the Italian medical exam, scoring 73%, it scored significantly lower in the French exam, with only 22%. The marked difference in ChatGPT’s performance between the Italian and French medical licensing exams likely reflects a combination of factors. The French exam’s multiple-answer question format posed a particular challenge, as language models like ChatGPT typically perform better on single-answer questions, such as those used in the Italian exam. Additionally, longer question lengths, especially in the French set, were linked to lower accuracy. Language-specific factors, including tokenization and training data exposure, also influenced results, while differences in national exam design philosophies likely contributed to the observed performance gap [1,80]. To summarize, this variability could be attributed to differences in exam formats, language subtleties, and subject matter, highlighting the need for localization and customization of AI models for different regions and medical systems.

The analysis of various in-training exams across multiple medical specialties demonstrates both the advantages and limitations of LLMs in the medical field. These show potential in medical training exams by showing their efficiency in processing large amounts of data, coupled with improved performance in certain domains, showing potential for enhancing clinical workflows by augmenting human decision-making. However, their performance varies across specialties, with notable limitations in problem-solving, nuanced reasoning, and explaining their logic. While ChatGPT-4 outperforms average human resident students in some cases, its accuracy is inconsistent, and it sometimes generates incorrect or misleading information [33]. These models can assist in medical education but still require human oversight, especially in complex decision-making areas.

A notable limitation identified in these models is their inability to handle questions involving figures and tables. This was demonstrated by studies on Japan’s National Medical Licensing Examination, where ChatGPT-4.0 scored 81.5%, passing the exam, while ChatGPT-3.5 only scored 42.8%. Both versions struggled with visual data, which is crucial for medical exams [40,81]. Additionally, ChatGPT-4 showed limited capability in interpreting medical images, achieving only 68% accuracy on image-based questions in another Japanese exam study [9]. While textual comprehension has improved, significant gaps remain in processing and interpreting visual information, which is essential for comprehensive medical practice. The study by Yang et al [85] evaluated the performance of ChatGPT-4V, ChatGPT-4, and GPT-3.5 Turbo on medical licensing exams involving images, showing GPT-4V’s high accuracy but significant limitations in image interpretation and explanation quality. While GPT-4V outperformed its predecessors in multiple-choice questions, its incorrect answers often featured poor image understanding and reasoning. The study highlights the need for further evaluation of GPT-4V’s capabilities before clinical integration [86].

The performance variability of ChatGPT-3.5 and GPT-4 across different countries and medical licensing exams can be attributed to several factors. Language nuances, such as regional variations in medical terminology, phrasing, and dialect, may affect how the AI interprets and responds to exam questions [80,87]. Exam formats also play a role, with different countries prioritizing multiple-choice questions, clinical scenarios, or essay-based responses, each requiring distinct approaches from the AI. Cultural differences in health care practices, ethical considerations, and medical education systems can influence how questions are framed and what knowledge is emphasized, affecting the AI’s ability to provide contextually appropriate answers. These factors combined can create significant variability in AI performance, highlighting the need for localized training and adaptation [87,85].

Despite significant advancements, ChatGPT’s performance still lags behind that of trained medical students. ChatGPT performed below the student average, with a score of 80.5/100 compared to the student average of 86.21/100 in the medical microbiology exam [79]. However, in some cases, such as the Peruvian National Licensing Medical Examination, GPT-4 outperformed human students, scoring 86% and surpassing 90% of human examinees [29]. These discrepancies suggest that while ChatGPT can achieve expert-level performance on standardized medical exams, it remains limited in handling complex, domain-specific tasks requiring deep understanding and critical thinking [82].

ChatGPT-4’s performance across different medical subjects further highlights its variability. In a study by Jang et al [16] on the Korean National Licensing Examination for Korean Medicine Doctors, ChatGPT-4 passed in 7 out of 12 subjects, excelling in herbology and neuropsychiatry but performing poorly in public health and acupuncture. It scored 85.9% on diagnosis-based questions, 63.2% on recall-based questions, and 53.5% on intervention-based questions, consistently outperforming ChatGPT-3.5. These results suggest the potential for AI models to specialize in certain medical fields, though challenges remain, especially with language nuances and culturally specific medical concepts [11,86,83,84].

While AI models provided mostly clinically valid information, they are not without flaws, particularly the risk of hallucinations or misinformation. Long et al [54] evaluated ChatGPT’s performance on otolaryngology exams, finding that it achieved a passing mark and showed higher accuracy with specific prompts. However, caution is advised due to the risk of AI-generated hallucinations or misinformation [33]. Similarly, Takagi et al [14] found ChatGPT-4 outperformed GPT-3.5 on the Japanese Medical Licensing Exam, but both faced challenges with hallucinations and inaccurate information [14]. Maitland et al [58] identified a key reason for these misinformation issues: language models are trained “to predict the next token in a sequence rather than verify factual accuracy.”

Concerns about AI misuse, such as cheating and misdiagnosis, remain significant in educational and clinical settings. A recent study highlighted the challenges posed by AI models like ChatGPT in providing relevant, readable, and accurate responses in medical exams [19,88,89]. The presence of multiple potentially correct answers poses a challenge for AI systems, as demonstrated in Kung et al’s [41] study, which compared ChatGPT’s performance to orthopedic surgery residents at various postgraduate levels. While AI models offer quick and accessible information, the risk of misuse and the need for further refinement cannot be overlooked. To address this issue, there should be guidelines and policies with clear ethical standards for AI usage. Transparency about AI decision-making should be ensured with accountability for misuse and error. Moreover, oversight committees must be formulated in each organization to monitor and review AI outputs and decision-making processes. There should be limited access to data with anonymization to protect privacy. Regular audits of AI models should be conducted for bias and inaccuracies, with diverse datasets being used. Training of staff on the ethical use of AI is necessary, with policies for responsible use. Validation of AI outputs with human experts’ assessments should be done with continuous feedback for the improvement of AI models [87,85].

The role of AI in generating medical exam questions and educational content also presents both opportunities and challenges. A multinational prospective study conducted by Cheung et al [25] highlighted how ChatGPT demonstrated the ability to generate medical graduate exam multiple-choice questions efficiently [25]. However, the AI’s questions lacked the depth, relevance, and specificity seen in human-generated content. This points to the nuanced complexities and limitations inherent in AI-driven content creation, including concerns about reliability, credibility, bias, and factual accuracy [33,90]. Thus, while ChatGPT shows potential, careful oversight is required to ensure its effective integration into medical education. Furthermore, the findings underline the growing potential that LLMs can play in medical education and licensing exams, but highlight the need for contextualization and adaptation, particularly in any assessments that involve visual information, native language perception, or abstracted clinical reasoning.

### Limitations

The study on ChatGPT-3.5 and 4.0 in medical education reveals several limitations. A primary concern is the models’ inability to interpret visual data, such as medical images, which are crucial for effective practice and examination performance. Additionally, the findings may lack generalizability across different medical disciplines. Potential biases in AI-generated content raise questions about reliability and equity in diverse educational contexts.

### Future Research and Gap

Further empirical data is essential to support the accuracy and utility of ChatGPT-3.5 and 4.0 in medical licensing and in-training examinations. While these versions show promising potential in processing vast amounts of medical knowledge, their application in critical, high-stakes environments requires rigorous validation. Current studies suggest variable performance, with both versions demonstrating strengths in general medical knowledge but limitations in reasoning and clinical decision-making. To solidify their role in medical education and assessment, more research is needed, especially focusing on advanced future iterations. These future versions must be scrutinized for improved understanding of complex medical scenarios, ethical considerations, and practical applications in real-world settings.

### Implications and Contributions

This study highlights the implications and contributions of ChatGPT versions by analyzing their accuracy in medical examinations across the globe. By synthesizing data from diverse studies conducted in various countries, it offers a broader perspective on the performance of ChatGPT-3.5 and 4.0 in medical licensing and in-training exams. The cross-country analysis showcases the potential of these AI models in adapting to different medical curricula and standards, contributing valuable insights into their applicability in international medical education. This global evaluation established a more comprehensive understanding of the strengths and limitations of ChatGPT, providing a foundation for further research and development in medical AI tools.

## Appendix

Multimedia Appendix 1 Funnel plot for publication bias: ChatGPT-4 vs ChatGPT-3.5 in medical licensing exams and residency exams.

Multimedia Appendix 2 PRISMA (Preferred Reporting Items for Systematic Reviews and Meta-Analyses) checklist.

## Abbreviations

AI: artificial intelligence
LLM: large language model
MLEs: medical licensing examinations
PRISMA: Preferred Reporting Items for Systematic Reviews and Meta-Analyses
ROBINS-I: Risk of Bias in Nonrandomized Interventional Studies
RR: risk ratio
USMLE: United States Medical Licensing Examination
