# Supplementary figures and images for: Evaluating the Potential and Accuracy of ChatGPT-3.5 and 4.0 in Medical Licensing and In-Training Examinations: Systematic Review and Meta-Analysis
Source: JMIR Med Educ. 2025 Sep 19;11:e68070. doi: 10.2196/68070 (PMC12495368; doi:10.2196/68070)

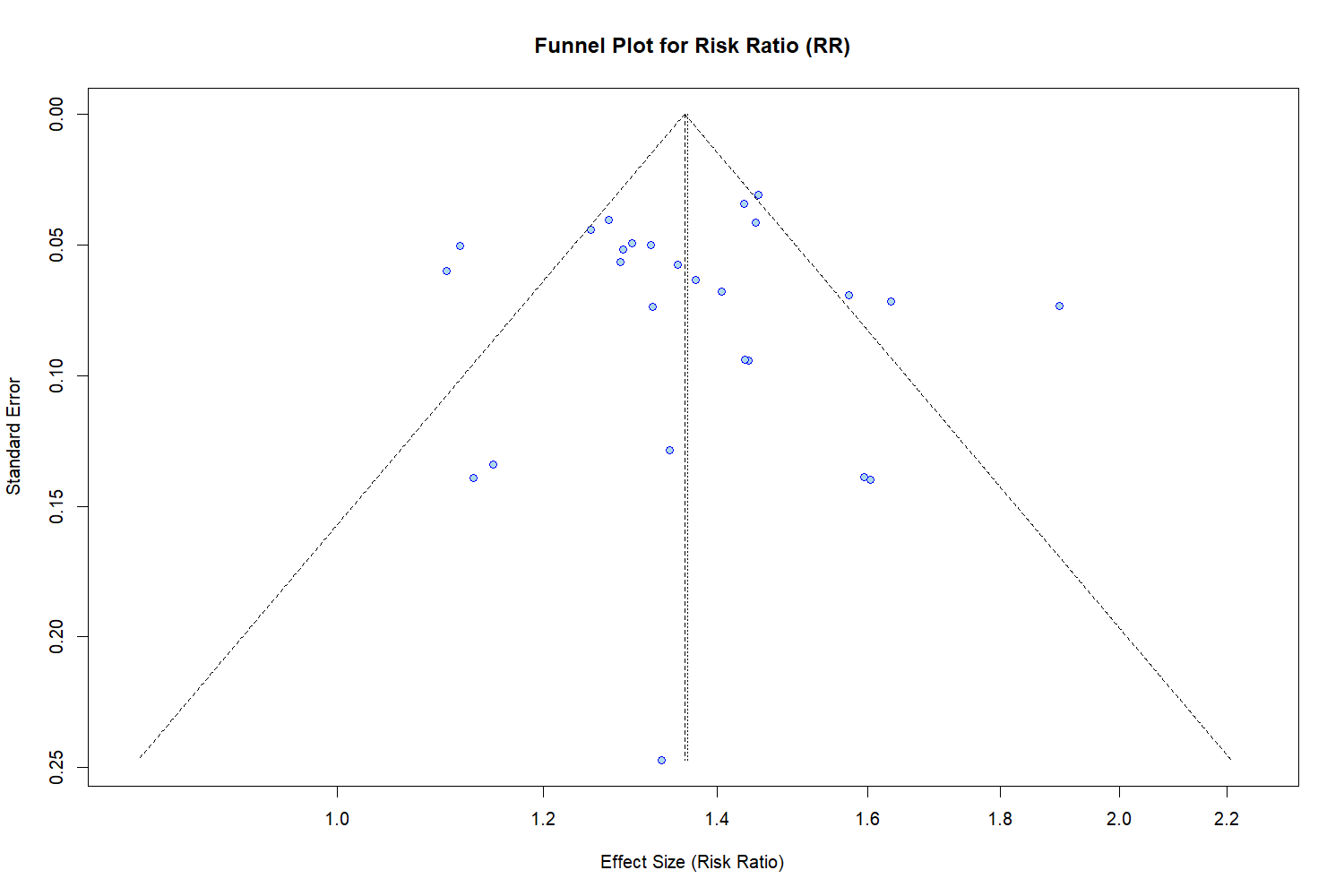

Supplement: Multimedia Appendix 1 [file mededu_v11i1e68070_app1.png]
